# Using computer-generated protein models to analyze mutations linked to Amelogenesis Imperfecta

**DOI:** 10.1371/journal.pone.0326679

**Published:** 2025-06-26

**Authors:** Nazlee Sharmin, Jerald Yuan, Ava K. Chow

**Affiliations:** Mike Petryk School of Dentistry, Faculty of Medicine and Dentistry, College of Health Sciences, University of Alberta, Edmonton, Alberta, Canada; Cholistan University of Veterinary and Animal Sciences, PAKISTAN

## Abstract

Amelogenesis Imperfecta (AI) is a disorder of tooth development caused by mutations in genes involved in several stages of tooth enamel formation. Few proteins involved in tooth development or developmental anomalies are explored in detail. Knowledge of 3D protein structure is essential to studying protein function. However, crystallized complete protein structures related to teeth and oral development are rare in the Protein Data Bank. Computational approaches for automated protein structure prediction have become a popular alternative for generating protein 3D structures. In this study, we aimed to explore the potential of using computer-generated protein models to analyze mutations linked to AI. We took a systematic approach to identify, screen, and analyze AI-linked protein variants. Proteins with AI-linked mutations were identified from the NCBI and OMIM databases, followed by screening of sequences for intrinsically disordered regions (IDRs). The iterative threading assembly refinement (I-TASSER) server was used to generate homology models for the wildtype and mutant proteins. PyMOL was used to analyze and compare the 3D structures of the proteins. Nineteen human genes with AI-associated mutations were identified from NCBI and OMIM. We identified multiple AI-associated protein variants with structural differences compared to their wildtype form. The current evidence aligns with several of the structural alterations identified in our study. Our findings suggest the potential of utilizing computer-generated protein models to investigate disease-associated mutations. However, careful consideration of models, templates, and alignments over the regions of interest is necessary to predict any potential structural impact of a disease-causing protein variant.

## Introduction

The development of a tooth and its surrounding structure is a complex and highly regulated biological process [1]. Advancements in molecular biology have revealed strict genomic and proteomic control of odontogenesis, which guides the position, number, size, and shape of our teeth [2]. More than 200 genes and their protein products have been identified in tooth development [3,4]. Consequently, it is not surprising that many developmental anomalies are found in teeth and the craniofacial regions that are linked to genetic mutations. Amelogenesis imperfecta (AI) is one such disorder of tooth development caused by mutations in genes involved in several stages of tooth enamel formation [5,6]. Amelogenesis, the process of enamel formation, involves several steps highly regulated by multiple genes and protein-protein interactions [6,7].

Few proteins involved in tooth and facial development or linked to oral developmental anomalies have been studied in detail. Knowledge of three-dimensional (3D) protein structure is essential to studying protein function [8,9]. However, crystallized, complete protein structures related to teeth and oral development are rare in the Research Collaboratory for Structural Bioinformatics (RCSB) Protein Data Bank (PDB, www.rcsb.org/) [10]. Obtaining a 3D protein structure using X-ray crystallography or nuclear magnetic resonance (NMR) spectroscopy is expensive, time-consuming, and not possible with all types of proteins [11]. A computational approach for automated protein structure prediction is a solution to this problem. Advancements in protein sequence alignment, detecting distant homologues, and modeling of loops and side chains have contributed to the reliable prediction of protein structure [12]. AlphaFold, for example, is a database of predicted protein structures using an artificial intelligence system developed by DeepMind [12]. In recent years, generative models have also been developed to design protein structures [13]. One example is RFdiffusion, which was developed by fine-tuning the RoseTTAFold structure prediction network for protein structure denoising tasks [13]. While databases like AlphaFold do not currently allow users to input custom protein sequences for structure prediction, several homology modeling tools like MODELLER, and SWISS-MODEL are available to predict the structure of a given protein sequence by aligning it with known template structures [14,15]. Each tool has its own advantages and limitations, depending on factors such as ease of use, accuracy, and template availability [16]. Although there are some reports of computer-generated protein models in drug discoveries [17,18], their applications in the study of disease-causing mutations are rare. In this study, we aimed to take an *in-silico* approach to study the mutations identified in AI. Our research questions were:

- What types of protein mutations have been identified as being associated with AI?
- How accurate are computational protein modeling tools in predicting the structural changes of proteins caused by mutations associated with AI?
- What is the potential of using computer-generated protein models to analyze mutations linked to AI?

## Materials and methods

### A. Identifying mutations associated with amelogenesis imperfecta through database search

**Fig 1:**
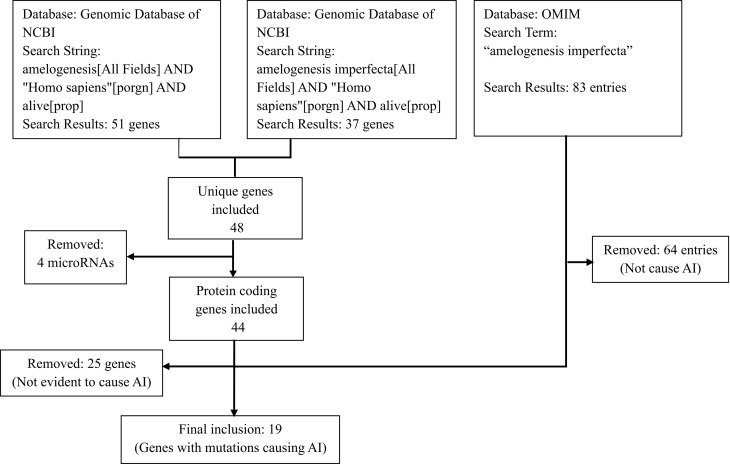
Flowchart of the search process to identify proteins with mutations involved in amelogenesis imperfecta (AI).

We took a systematic approach to search the genomic database of the National Library of Medicine (NCBI) with two terms, ‘Amelogenesis Imperfecta’ AND ‘Homo sapiens, to identify human genes and mutations involved in AI [19]. We further searched the same database with another two terms, ‘Amelogenesis’ AND ‘Homo sapiens.’ The two-way search aimed to identify all possible genes involved in AI, with and without a role in enamel formation. Non-protein-coding microRNAs and genes with no reported mutations were excluded. Additionally, the database of Online Mendelian Inheritance in Man (OMIM) was searched with the keyword “amelogenesis imperfecta” [20]. The finally included genes were those with AI-causing mutations, as documented in NCBI and OMIM. No additional database filters and pathogenicity thresholds were applied. The details of our search string and screening process are outlined in Fig 1.

### B. Screening for intrinsically disordered proteins (IDPs)

In recent years, there has been a growing recognition that a large fraction of the human proteome is intrinsically disordered, meaning that these proteins carry out their biological functions without attaining a stable 3D structure under physiological conditions [21]. While intrinsically disordered proteins (IDPs) present unique challenges for predicting their 3D structures, several algorithms are available that can predict the disorder within a given protein sequence [22]. To screen our selected proteins for intrinsically disordered regions (IDR), D2P2 server was used, which provides IDP/IDR predictions made by 9 different predictors across 1765 complete genomes containing 10,429,761 sequences from 1256 distinct species [23].

### C. Selecting protein modeler and evaluation tools for homology models

To select a protein modeler to produce reliable, full-length models for both the wildtype and mutant proteins, several test protein sequences, with and without homologous templates were ran in several modelers, including the iterative threading assembly refinement server (I-TASSER), SWISS-MODEL, Robetta, and PEP-FOLD3. SWISS- MODEL did not predict the structure of the template-independent regions of the custom protein sequence. The *de novo* protocol for Robetta is optimized for <120 residues single-domain proteins [24]. Structure prediction by PEP-FOLD was limited to only between 5 and 50 amino acid residues [25]. The I-TASSER was chosen over those modelers, considering the length and the quality of the output. Besides template dependence, I-TASSER applies *ab initio* structure modeling for protein targets in the regions with no or weakly homologous templates [26]. Several models from AlphaFold were used in this study to compare with the wildtype protein, but as AlphaFold does not allow users to input sequences, it was not used to create models for the mutant proteins [12]. Substitution/missense, and in-frame deletion mutations were modelled with I-TASSER.

I-TASSER returned five predicted full-length protein tertiary structures for each sequence input with corresponding C-scores, TM-scores, and the root-mean-square distance (RMSD) values. The C-score is calculated from the significance of threading template alignments and the convergence parameters of the structure assembly simulations. Higher values for C-scores represent higher confidence for the predicted protein model. TM-score and RMSD are estimated based on C-score and protein length following the correlation observed between these qualities [26]. For each of our target proteins, the best-predicted model was chosen based on the C-score, TM-score and RMSD.

To further evaluate the quality of the mutant and wildtype protein models generated by I-TASSER, the ProSA program (Protein Structure Analysis) and Ramachandran plot were used. ProSA-web analyzes a given protein structure and calculates a Z score (≤10), measuring the deviation of the total energy of the structure from an energy distribution derived from random conformations [27]. VADAR (Volume Area Dihedral Angle Reporter), a comprehensive web server, was used to generate the Ramachandran plot to get a visual assessment of protein structure quality [28].

### D. Analysis of the protein structures using PyMOL

PyMOL was used to analyze and compare our wildtype and mutant 3D proteins [29]. To identify structural alterations in the protein’s backbone the wildtype protein models were superimposed on each of its varients using the sequence-independent ‘Super’ algorithm of PyMOL. The electrostatic potential surfaces of the wildtype and mutant proteins were also analyzed to determine any change in the surface charge between the mutant and the wildtype proteins.

## Results

### A. Proteins with mutations involved in amelogenesis imperfecta (AI)

Searching the database with ‘Amelogenesis Imperfecta’ AND ‘Homo sapiens’ and ‘Amelogenesis’ AND ‘Homo sapiens’ returned 37 and 51 genes, respectively. Four microRNAs were excluded from the list, leaving 44 unique protein-coding genes after the first round of screening. Searching the OMIM database returned 83 entries with “amelogenesis imperfecta”, of which 64 were removed for not being related to AI. After the 2^nd^ round of screening and comparison between databases, as of March 2025, 19 genes were included in our study reported to have mutations in patients, documented in NCBI and in OMIM. For the finally included genes, data related to HGNC gene symbol, UniProtKB identifier, UniProtKB Annotation score cytogenetic location, protein and its function in tooth development, dental disease (OMIM phenotype), mutations (OMIM variants), and OMIM identifier were collected from NCBI, OMIM, and published literature. (Table 1).

**Table 1:** Human genes with known association with Amelogenesis Imperfecta (AI).

Genes associated with Amelogenesis Imperfecta (AI)				Encoded Proteins		Dental Disease		
Gene symbol (HGNC): Gene name	Genbank Identifier	UniProtKB identifier (Annotation score)	Cytogenetic location	Protein name	Protein function in tooth development	OMIM phenotype(s)	OMIM identifier	OMIM Variant(s)
LAMB3: Laminin subunit beta 3	3914	Q13751 (Annotation score: 5)	1q32.2	Laminin subunit beta-3 precursor	Differential expression of laminin chains in periodontal ligament (PDL) of deciduous and permanent teeth was found, suggesting an involvement of laminin-dependent pathways in the control of physiological differences between them [30]	Amelogenesis Imperfecta, Type IA	150310	Frameshift: 8-BP DEL, NT3446 1-BP INS, 3392G Nonsense/ Truncation mutation: S1144X
ITGB6: integrin subunit beta 6	3694	P18564 (Annotation score: 5)	2q24.2	Integrin beta-6 isoform a precursor	TGF-β1 is reported to be involved in a complicated dynamic interaction with matrix metalloproteinases (MMPs) and/or dentin sialophosphoprotein (DSPP)-derived proteins present in dental pulp, odontoblasts and dentin. [31]	Amelogenesis imperfecta, type IH	147558	Substitution/ Missense mutation: A143T, H275Q, P196T, A143T + H275Q Nonsense/ Truncation mutation: R616X, P196T + R616X
AMTN: Amelotin	401138	Q6UX39 (Annotation score: 5)	4q13.3	Amelotin isoform 1 precursor	Amelotin is expressed in the maturation-stage of ameloblasts. [32]	Amelogenesis imperfecta, type IIIB	610912	In-frame deletion: 8,678 bp deletion
AMBN: Ameloblastin	258	Q9NP70 (Annotation score: 5)	4q13.3	Ameloblastin precursor	The encoded protein may be important in enamel matrix formation and mineralization [32].	Amelogenesis imperfecta, type IF	601259	In-frame deletion: 2,347 bp deletion Splicing Mutation: c.532-1G-C
ENAM: Enamelin	10117	Q9NRM1 (Annotation score: 5)	4q13.3	Enamelin isoform 1 precursor	The protein is involved in the mineralization and structural organization of enamel [32]	Amelogenesis imperfecta, type IB Amelogenesis imperfecta, type IC	606585	Nonsense/ Truncation mutation: K53X Frameshift: P422fsX448 Splicing Mutation: c.841 + 1G-A c.534 + 1G-A
ODAPH: Odontogenesis Associated Phosphoprotein	152816	Q17RF5 (Annotation score: 4)	4q21.1	Odontogenesis associated phosphoprotein isoform 2 precursor	Synthetic peptide corresponding to the phosphorylated C terminus of C4ORF26 can promote hydroxyapatite nucleation and support crystal growth. [33]	Amelogenesis imperfecta, type IIA4	614829	Nonsense/ Truncation mutation: R77X, W106X, C43X In-frame deletion/ insertion: 6-BP DEL/16-BP INS Splicing Mutation: Mutation in the splice acceptor site of intron 1.
SLC10A7: Solute carrier family 10 member 7	84068	Q0GE19 (Annotation score: 5)	4q31.22	Sodium/bile acid cotransporter 7 isoform b	Slc10a7 transcripts were expressed in the epithelium of the developing mouse tooth, bones undergoing ossification. [34]	Amelogenesis Imperfecta (Short stature, skeletal dysplasia with scoliosis)	611459	Substitution/ Missense mutation: L74P, G130R Splice site mutaion VS8AS, A-G, −16 Nonsense/ Truncation mutation: Q172X
FAM83H: Family with sequence similarity 83 member H	286077	Q6ZRV2 (Annotation score: 5)	8q24.3	FAM83H	Studies indicate that the Fam83h mutation could inhibit the mineralization in ameloblasts by activating Wnt/β-catenin signaling pathway [35].	Amelogenesis imperfecta, type IIIA	611927	Nonsense/ Truncation mutation: R325X, Q398X, E415X, Y297X, W460X, Q677X, Q456X, Q470X, S287X, E694X Frameshift: 2-BP DEL, 1872CC (Leu625fsTer703) 2-BP DEL, 923TC (Leu308fsTer323)
RELT: RELT TNF receptor	84957	Q969Z4 (Annotation score: 5)	11q13.4	Tumor necrosis factor receptor superfamily member 19L precursor	Relt-/- mice generated by CRISPR/Cas9 exhibited incisor and molar enamel malformations. Relt-/- enamel had a rough surface and underwent rapid attrition [36].	Amelogenesis imperfecta, type IIIC	611211	Substitution/ Missense mutation: R422P Frameshift: 2-BP DEL, 1169CT (Pro390fsTer35). Slice site mutation: IVS3AS, A-G, −2
MMP20: Matrix metallopeptidase 20	9313	O60882 (Annotation score: 5)	11q22.2	Matrix metalloproteinase-20 preproprotein	MMP20 could degrade amelogenin. They suggested that MMP20 plays a central role in tooth enamel formation, as the recombinant MMP20 is found to degrade amelogenin. [37]. ^28^	Amelogenesis imperfecta, type IIA2	604629	Substitution/ Missense mutation: H226Q, H204R Nonsense/ Truncation mutation: W34X Slice site mutation: IVS6AS, A-T, −2*
GPR68: G protein-coupled receptor 68	8111	Q15743 (Annotation score: 5)	14q32.11	Ovarian cancer G-protein coupled receptor 1	Studies suggest a possible role for GPR68 as a pH monitor of the developing enamel matrix [38].	Amelogenesis imperfecta, hypomaturation type, IIA6	601404	In-frame deletion 450-BP DEL, NT386 Frameshift: 2-BP DEL, NT667 (Lys223GlyfsTer113) Substitution/ Missense mutation: L74P
SLC24A4: Solute carrier family 24 member 4	123041	Q8NFF2 (Annotation score: 5)	14q32.12	Sodium/potassium/calcium exchanger 4 isoform 1 precursor	Wang et al., (2014) performed immunohistochemical analyses of developing teeth of mice and found that Slc24a4 was expressed in maturation-stage of the ameloblasts [39].	Amelogenesis imperfecta, type IIA5	609840	Substitution/ Missense mutation: A146V, S499C Nonsense/ Truncation mutation: R339X
WDR72: WD repeat domain 72	256764	Q3MJ13 (Annotation score: 5)	15q21.3	WD repeat-containing protein 72 isoform a	WDR72 plays an important role in enamel mineralization, possibly due to endocytic vesicle trafficking [40].	Amelogenesis imperfecta, type IIA3	613214	Nonsense/ Truncation mutation: W978X, S783X Frameshift: 2-BP DEL, 1467AT 1-BP DEL, 2857A
DLX3: distal-less homeobox 3	1747	O60479 (Annotation score: 5)	17q21.33	Homeobox protein DLX-3	Pang et al., (2020) used dlx3b mutant zebrafish for this study, where dlx3b-/- group as compared with the dlx3b + / + group. Scanning electron microscopy study showed morphological surface changes in pharyngeal teeth enameloid, with a decrease in the mineral content. Specific secretory calcium-binding phosphoprotein genes were significantly downregulated in dlx3b mutants [41].	Amelogenesis imperfecta, type IV Trichodontoosseous syndrome	600525	Frameshift: 2-BP DEL, 560CT
FAM20A: FAM20A golgi associated secretory pathway pseudokinase	54757	Q96MK3 (Annotation score: 5)	17q24.2	Pseudokinase FAM20A isoform a precursor	Fam20a transcripts showed expression within the secretory ameloblasts, stratum intermedium cells, and the underlying analog of the stratum intermedium [42].	Amelogenesis imperfecta, type IG (enamel-renal syndrome)	611062	Nonsense/ Truncation mutation: R136X, R478X Frameshift: 2-BP DEL, 34CT 1-BP DEL, 612C 5-BP DEL,NT1175; Slice site mutation: IVS4AS, A-G, −2
ACP4: Acid phosphatase 4	93650	Q9BZG2 (Annotation score: 5)	19q13.33	Testicular acid phosphatase precursor	Immunohistochemical studies showed that ACPT was localized in ameloblasts, follicular cells, odontoblasts, and osteoblasts, but not in maturation-stage ameloblasts, indicating that ACPT functions during the secretory stage of amelogenesis [43].	Amelogenesis imperfecta, type IJ	606362	Substitution/ Missense mutation: S238L, R111C, R76C, A128P, E133K
KLK4: Kallikrein related peptidase 4	9622	Q9Y5K2 (Annotation score: 5)	19q13.41	Kallikrein-4 isoform 1 preproprotein	Studies indicate that KLK4 function independently, but are necessary for proper enamel maturation [44].	Amelogenesis imperfecta, type IIA1	603767	Nonsense/ Truncation mutation:W153X Frameshift: 1-BP DEL, 245G
AMELX: Amelogenin X-linked	265	Q99217 (Annotation score: 5)	Xp22.2	Amelogenin, X isoform 3 precursor	Amelogenins are highly conserved proteins secreted by ameloblasts, and constitute 90% of the enamel organic matrix [45].	Amelogenesis imperfecta, type 1E	300391	Frameshift 1-BP DEL, 155C 1-BP DEL, 473C 1-BP DEL, 541C 1-BP DEL, 420C 5KB DEL 96-KB DEL In-frame deletion: 9-BP DEL Substitution/ Missense mutation: T51I, P70T, W4S, M1T Nonsense/ Truncation mutation: E191X
Sp6: Sp6 transcription factor	80320	Q3SY56 (Annotation score: 5)	17q21.32	Transcription factor Sp6	SP6 belongs to a family of transcription factors that bind to GC-rich sequences and related GT and CACCC boxes [46].	Amelogenesis imperfecta, type IK	608613	Substitution/ Missense mutation: A273K A273M

The proteins encoded by these 19 genes are classified as enamel matrix proteins (amelogenin, ameloblastin, enamelin), enamel matrix proteases (matrix metallopeptidases, KLK4), cell-cell and cell-matrix adhesion proteins (integrin, laminin, amelotin, FAM83H), transport proteins (WD repeat domain 72, solute carrier family 24), proteins involved in pH sensing, crystal nucleation, and unknown functions (G-protein-coupled receptor 68, RELT TNF receptor, odontogenesis associated phosphoprotein, distal-less homeobox 3, acid phosphatase 4, FAM20A, Solute carrier family 10 member 7, Sp6 transcription factor) [6]. An analysis of 82 mutants are grouped as frameshift (20), truncation (26), substitution (24), splice-site mutation (7), and in-frame deletion/insertion (5). (Table 1).

Three truncation and 2 deletion mutations was identified as candidates for inducing Nonsense-mediated mRNA decay (NMD) (Table 2). NMD is a quality-control mechanism in eukaryotes that screens newly synthesized mRNAs and degrades the ones with premature termination codons to prevent the formation of disease-causing truncated proteins [47].

**Table 2:** OMIM variants (Mutations) likely to induce Nonsense-mediated mRNA decay (NMD).

OMIM variants	Gene	Associated OMIM phenotype(s)
K53X	ENAM: Enamelin	Amelogenesis imperfecta, type IB A heterozygous c.438A-T transversion in exon 4 of the ENAM gene was identified in patients with type IB AI. This nonsense mutation results in a truncated peptide of 52 amino acid, where the full-length Enamelin protein is 1142 amino acids long [48].
C43X	ODAPH: Odontogenesis Associated Phosphoprotein	Amelogenesis imperfecta, type IIA4 In a family with hypomineralized amelogenesis imperfecta (AI type IIA4) a homozygous 129C-A transversion was identified in the C4ORF26 gene, predicted to cause immature truncation of loss of function of the protein [33].
W34X	MMP20: Matrix metallopeptidase 20	Amelogenesis imperfecta, type IIA2 In a child with hypoplastic amelogenesis imperfecta and wide overbite, a homozygous 102G-A transition in the MMP20 gene was identified, resulting in a trp34-to-ter (W34X) substitution in exon 1 of the protein [49].
2-BP DEL, 34CT	FAM20A: FAM20A golgi associated secretory pathway pseudokinase	Amelogenesis imperfecta, type IG (enamel-renal syndrome) In a patient with amelogenesis imperfecta and gingival fibromatosis, homozygosity for a 2-bp deletion (c.34_35delCT) in exon 1 of the FAM20A gene was identified. The mutation resulted in a frameshift and a premature termination of the protein [50].
1-BP DEL, 155C	AMELX: Amelogenin X-linked	Amelogenesis imperfecta, type 1E In a family with X-linked amelogenesis imperfecta, a deletion of one cytosine in exon 5 of the AMELX gene was identified, resulting in an alteration of the reading frame and introduction a stop codon [51].

### B. Proteins with Intrinsically disordered regions (IDRs)

Five proteins reported no IDRs. High (>50%) IDRs were identified in six proteins. Proteins with less than 30% IDRs were used for 3D structure prediction and further analysis (Table 3).

**Table 3:** Screening Protein Sequence for Intrinsically disordered regions (IDRs).

		Results from D2P2: Database of Disordered Protein Predictions	
Gene symbol (HGNC): Gene name	Protein name	The bold portions of sequence are where there is 75% agreement between all predictors in the database for this region being disordered.	Percent of disordered region
LAMB3: Laminin subunit beta 3	Laminin subunit beta-3 precursor	>NP_001017402.1 MRPFFLLCFALPGLLHAQQACSRGACYPPVGDLLVGRTRFLRASSTCGLTKPETYCTQYGEWQMKCCKCDSRQPHNYYSHRVENVASSSGPMRWWQSQNDVNPVSLQLDLDRRFQLQEVMMEFQGPMPAGMLIERSSDFGKTWRVYQYLAADCTSTFPRVRQGRPQS**W**QDVRCQSLPQRPNARLNGGKVQLNLMDLVSGIPATQSQKIQEVGEITNLRVNFTRLAPVPQRGYHPPSAYYAVSQLRLQGSCFCHGHADRCAPKP**GASA**GPSTAVQVHDVCVCQHNTAGPNCERCAPFYNNRPWRPAEGQDAHECQRCDCNGHSETCHFDPAVFAASQGAYGGVCDNCRDHTEGKNCERCQLHYFRNRRPGASIQETCISCECDPDGAVPGAPCDPVTGQCVCKEHVQGERCDLCKPGFTGLTYANPQGCHRCDCNILGSRRDMPCDEESGRCLCLPNVVGPKCDQCAPYHWKLASGQGCEPCACDPHNSLSPQCNQFTGQCPCREGFGGLMCSAAAIRQCPDRTYGDVATGCRACDCDFRGTEGPGCDKASGRCLCRPGLTGPRCDQCQRGYCNRYPVCVACHPCFQTYDADLREQALRFGRLRNATASLWSGPGLEDRGLASRILDAKSKIEQIRAVLSSPAVTEQEVAQVASAILSLRRTLQGLQLDLPLEEETLSLPRDLESLDRSFNGLLTMYQRKREQ**FE**KI**S**SADPSGAFR**M**LSTAYEQS**A**QAAQQV**SDSS**RLLDQLRDSRREAERL**VRQAGGGG**GTG**SP**KLVALRLEMSSLPDLTPTFNKLCGNSRQMACTPISCPGELCPQDNGTACGSRCRGVLPRAGGAFLMAGQVAEQLRGFNAQLQRTRQMIRAAEESASQIQS**SAQRLETQVSASRS**QMEEDVRRTRLLIQQVRDFLTDPDTDAATIQEVSEAVLALWLPTDSATVLQKMNEIQAIAARLPNVDLVLSQTKQDIARARRLQAEAEE**ARSRA**HAVEGQVEDVVGNLRQGTVALQEAQDTMQGTSRSLRLIQDRVAEVQQVLRPAEKLVTSMTKQLGDFWTRMEELRHQARQQGAEAVQAQQL**AEGA**S**EQ**ALS**A**QEGFERIKQKYAELKDRLGQSSML**GE**QG**A**RIQSVKTEAEELFGETMEMMDRMKDMELELLRGSQAIMLRSADLTGLEKRVEQIRDHINGRVLYYATCK	Sequence: 1172 aa Predicted IDR: 44 Percent of disordered region: 3.75% (Included)
ITGB6: integrin subunit beta 6	Integrin beta-6 isoform a precursor	No agreed upon disordered regions found.	(Included)
AMTN: Amelotin	Amelotin isoform 1 precursor	>NP_997722.1 MRSTILLFCLLGSTRSLPQLK**PALGLPPTKLAPDQGTLPNQQ**QSNQVFPSLSLIPLTQMLTLGPDLHLLNPAA**GMTPGTQT**HPLTLGGLNVQQQLHPHVLPIFVTQLGAQGTILSSEELPQIFTSLIIHSLFPGGIL**PTSQAGANPDVQDGSLPAGGAGVNPATQGTPAGRLPTPSGTDDDFAVTTPAGIQRSTHAIEEATTESANGIQ**	Sequence: 209 aa Predicted IDR: 101 Percent of disordered region: 48.3%
AMBN: Ameloblastin	Ameloblastin precursor	>NP_057603.1 MSASKIPLFKMKDLILILCLLEMSFAVPFFPQQSGTPGMASLSLETMRQLGSLQRLNTLSQYSRYGFGKSFNSLWMHGLLPPHSSLP**WMRPREH**ET**QQYEYSLPVHPPPLPSQPSLKPQQPGLKPFLQSAAA**T**TNQA**TA**LK**E**ALQPPIHLGHLPLQEGELPLVQQQVAPSDKPPKPELPGVDFADPQGPSLPGMDFPDPQGPSLPGLDFAD**PQGSTIFQIARLISHGP**MPQN**K**Q**SPLYPGMLYVPFGANQLNAPA**RLG**I**MSSEEVAGGREDPMA**YGAMFPGFGGMRPGFEGMPHNP**AMGGDFT**L**EFDSPVAATKGPENEEGGAQGSPMPEANPDNLENPAFLTELEPAPHAGLLALPKDDIPGLPRSPSGKMKGLPSVTPAAADPLM**TPELADVYRTYDADMTTSVDFQ**EEATMDTTMAPNSLQTSMPGNKAQEPEMMHDAWHFQEP**	Sequence: 447 aa Predicted IDR: 276 Percent of disordered region: 61.7%
ENAM: Enamelin	Enamelin isoform 1 precursor	>NP_114095.2 MLVLRCRLGTSFPKLDNLVPKGKMKILLVFLGLLGNSVAMPM**HMPRMPGFSSK**SEEMMRYNQFNFMNGPHMAHLGPF**F**GNG**L**PQQFPQY**QMPMWPQPPPNTWHPRKSSAPKRHNKTDQTQETQKPNQTQSKKPPQKRPLKQPSHNQPQPEEEAQPPQAFPPFGNG**LFPYQQPPW**QIPQRLPPPGYGRPPISNEEGG**NPYFGYFGYHGFGGRPPYY**SEEMFEQDFEKPKEEDPPKAESPGTEPTANSTVTETNSTQPNPKGSQGGNDTSPTGNSTPGLNTGNNPPAQNGIGPLPAVNASGQGGPGSQIPWRPSQPNIRENHPYPNIR**NFPSGRQWYFTGTVMGHR**QNR**PF**YRNQQ**VQRGPRWNFFAWERKQVARPG**NPVY**HKAYP**PTSRGNYPNYAGNPANLRRKPQGPNKHPVGTTVAPLGPKPGPVVRNEKIQNPKEKPLGPKEQIIVPTKNPTSPWRNSQQYEVNKSNYKLPHSEGYMPVPNFNSVDQHENSYYPRGDSRKVPNSDGQTQSQNLPKGIVLGSRRMPYESETNQSELKHSSYQPAVYPEEIPSPAKEHFPAGRNTWDHQEISPPFKEDPGRQEEHLPHPSHGSRGSVFYPEYNPYDPRENSPYLRGNTWDERDDSPNTMGQKESPLYPINTPDQKEIVPYNEEDPVDPTGDEVFPGQNRW**GEELSFK**GGP**TVRHYEGE**QYTSNQP**KEYLPYS**LDN**PSKPREDFYYSEFYPWSPDENFPSYNTAS**TMPPPIESRG**Y**YVN**N**A**A**GP**EESTLFPSRNSWD**HRIQAQGQRERRP**Y**F**NRNIWDQATH**LQKAPARPPDQKGNQPYYSN**TPAGLQKNPIWHEGENLNYGM**QITRMNSP**ERE**HSSFPNFI**PPSYPSGQKEAHLFHLSQRGS**CCAGSSTGPKDNPLALQDYTPSYGLAPGENQDTSPLYTDGSHTKQTRDIISPTSILPGQRNSSEKRESQNPFRDDVSTLRRNTPC**SIKN**QLGQKEIMPFPEASSLQSKNTP**CLKNDLGGDGN**NIL**EQVFEDNQLNERTVDL**TPEQLVIGTPDEGSNPEGIQSQVQENESERQQQRPSNI**LHLPCFGSKL**AKHHSSTTGTPSSDGRQSPFDGDSITPTENPNTLV**ELATEEQFKSI**NVDPLD**ADEHSPFEFLQRGTNVQDQVQDCLLLQA	Sequence: 1142 aa Predicted IDR: 783 Percent of disordered region: 68.5%
ODAPH: Odontogenesis Associated Phosphoprotein	Odontogenesis associated phosphoprotein isoform 2 precursor	>NP_848592.2 MARRHCFSYWLLVCWLVVTVAEGQEE**VFTPPGDSQ**NNA**DA**TDCQIFTLTPPPAPR**SPVTR**AQPITKTPRCPFHFFPRRPRIHFRFPNRPFVPSRCNHRFPFQPFYWPHRYLTYRYFPRRRL**Q**R**GSSSEES**	Sequence: 130 aa Predicted IDR: 23 Percent of disordered region: 17.6% (Included)
SLC10A7: Solute carrier family 10 member 7	Sodium/bile acid cotransporter 7 isoform b	No agreed upon disordered regions found.	(Included)
FAM83H: Family with sequence similarity 83 member H	FAM83H	>NP_940890.4 **MARRSQSSSQGDNPLAPG**YLPPHYKEYYRLAVDALAEGGSEAYSRFLATEGAPDFLCPEELEHVSRH**LRPPQYVTREPPEG**SLLDVDMDGSSGTYWPVNSDQAVPELDLGWPLTFGFQGTEVTTLVQP**PPPDSPS**IKDEARRMIRSAQQVVAVVMDMFTDVDLLSEVLEAAARRVPVYILLDEMNAQHFLDMADKCRVNLQHVDFLRVRTVAGPTYYCRTGKSFKGHVKEKFLLVDCAVVMSGSYSFMWSFEKIHRSLAHVFQGELVSSFDEEFRILFAQSEPLVPSAAALARMDAYALAPYAGAGPLVGVPGVGAPTPFSFPKRAHLLFPPPREEGLGFPSFLDPDRHFLSAFRREE**PPRMPGGALEPHAGLRPLSRRLEAEAG**PAGELAGARGFFQARHLEMDAFKRHSFATEGAGAVENFAAARQVSRQTFLSHGDDFRFQTSHFHRDQL**YQQQYQWDPQLTPARPQGL**FEK**LRGG**RAGF**ADPDDFTLGAGPRFPELGPDGHQRLDYVPSSASREVRHGSDPAFAPGPRGLEPSGAPRPNLTQRFPCQAAARPGPDPAPEAEPERRGGPEGRAGLR**RWRLASYLSG**CHGEDGGDDGLPAPMEAEAYEDDVLAPGGRAPAGDLLPSAFRVPAAFPTKVPVPGPGSGGNGPEREGPEEPGLAKQDSFRSRLNPL**VQRSSRLRSS**LIFS**TSQ**AE**GAAG**AA**AATEKVQLLHKE**QTVSETLGPGGEAVRSAAS**TKVAELLE**KYKGPARDPGGGAGAIT**VASHSKAVVSQAWREE**VAAPGAVGGE**RR**S**LESCLLDLRDS**FAQQLHQEAERQPGAASLTAAQLLDTLGRSGSDRLPSRFLSAQSHSTSPQGLDSPLPLEGSGAHQVLHNESKGSPTSAYPERKGSPTPGFSTRRGSPTTGFIEQKGSPTSAYPERRGSPVPPVPERRSSPVPPVPERRGSLTLTISGESPKAGPAEEGPSGPMEVLRKGSLRLRQLLSPKGERRMEDEGGFPVPQENGQPESPRRLSLGQGDSTEAATEERGPRARLSSATANALY**SSNLRDDTKA**IL**E**QISAHGQKHRAVPAPSPGPTHNSPELGRPPAAGVLAPDMSDK**DKCSAIFR**SD**SLG**TQGRLSRTLPASAEERDRLLRRMESMR**KEKRVYSRFEVFCKK**EEASSPGAGEGPAEEGTRDSKV**GKFVPKILGTFKSKK	Sequence: 1179 aa Predicted IDR: 654 Percent of disordered region: 55.4%
RELT: RELT TNF receptor	Tumor necrosis factor receptor superfamily member 19L precursor	>NP_689408.1 MKPSLLCRPLSCFLMLLPWPLATLTSTTLWQCPPGEEPDLDPGQGTLCRPCPPGTFSAAWGSSPCQPHARCSLWRRLEAQVGMATRDTLCGDCWPGWFGPWGVPRVPCQPCSWAPLGTHGCDEWGRRARRGVE**VAAGASSGGETRQPGNGTRAGGPE**ETAAQYAVIAIVPVFCLMGLLGILVCNLLKRKGYHCTAHKEV**GPGPGGGGSGINPAYRT**EDANEDTIGVLVRLITEKKENAAALEELLKEYHSKQLVQ**TSHRPVSKLPPAPPNVPH**ICPHRHHLHTVQGLASLSGPCCSRCSQKKWPEVLLSPEAV**AATTPVPSLLPNPTRVPKAGAKAG**RQGEITILSVGRFRVARIPEQRT**SSMVSEVKTITEAGPSWGDLPDSPQPGLPPEQQALLGSGGSRTKWLKPPAENK**AE**EN**RYVVRLSESNLVI	Sequence: 430 aa Predicted IDR: 138 Percent of disordered region: 32.1%
MMP20: Matrix metallopeptidase 20	Matrix metalloproteinase-20 preproprotein	No agreed upon disordered regions found.	(Included)
GPR68: G protein-coupled receptor 68	Ovarian cancer G-protein coupled receptor 1	>NP_001171147.1 MGNITADNSSMSCTIDHTIHQTLAPVVYVTVLVVGFPANCLSLYFGYLQIKARNELGVYLCNLTVADLFYICSLPFWLQYVLQHDNWSHGDLSCQVCGILLYENIYISVGFLCCISVDRYLAVAHPFRFHQFRTLKAAVGVSVVIWAKELLTSIYFLMHEEVIEDENQHRVCFEHYPIQAWQRAINYYRFLVGFLFPICLLLASYQGILRAVRRSHGTQKSRKDQIQRLVLSTVVIFLACFLPYHVLLLVRSVWEASCDFAKGVFNAYHFSLLLTSFNCVADPVLYCFVSETTHRDLARLRGACLAFLTCSRTGRAREAY**PL**GAP**EASGKSGAQGEE**PEL**LTKLHPAF**QTP**NSPGSGGFPTGR**LA	Sequence: 365 aa Predicted IDR: 34 Percent of disordered region: 9.3% (Included)
SLC24A4: Solute carrier family 24 member 4	Sodium/potassium/calcium exchanger 4 isoform 1 precursor	>NP_705932.2 MALRGTLRPLKVRRRREMLPQQVGFVCAVLALVCCASGLFGSLGHKTASASKRVLPDTWRNRKLMAPVNGTQTAKNCTDPAIHEFPTDLFSNKERQHGAVLLHILGALYMFYALAIVCDDFFVPSLEKICERLHLSEDVAGATFMAAGSSTPELFASVIGVFITHGDVGVGTIVGSAVFNILCIIGVCGLFAGQVVRLTWWAVCRDSVYYTISVIVLIVFIYDEQIVWWEGLVLIILYVFYILIMKYNVKMQAFFTVKQKSIANGNPVNSELEAGNDFYDGSYDDPSVPLLGQVKEKPQYGKNPVVMVDEIMSSSPPKFTFPEAGLRIMITNKFGPRTRLRMASRIIINERQRLIN**SANGVSSKPLQNGRHENIENGNVPVENPEDPQQNQEQQPPPQPPPPEPEPVEADFL**SPFSVPEARGDKVKWVFTWPLIFLLCVTIPNCSKPRWEKFFMVTFITATLWIAVFSYIMVWLVTIIGYTLGIPDVIMGITFLAAGTSVPDCMASLIVARQGLGDMAVSNTIGSNVFDILVGLGVPWGLQTMVVNYGSTVKINSRGLVYSVVLLLGSVALTVLGIHLNKWRLDRKLGVYVLVLYAIFLCFSIMIEFNVFTFVNLPMCREDD	Sequence: 622 aa Predicted IDR: 56 Percent of disordered region: 9% (Included)
WDR72: WD repeat domain 72	WD repeat-containing protein 72 isoform a	>NP_877435.3 MRTSLQAVALWGQKAPPHSITAIMITDDQRTIVTGSQEGQLCLWNLSHELKISAKELLFGHSASVTCLARARDFSKQPYIVSAAENGEMCVWNVTNGQCMEKATLPYRHTAICYYHCSFRMTGEGWLLCCGEYQDVLIIDAKTLAVVHSFRSSQFPDWINCMCIVHSMRIQEDSLLVVSVAGELKVWDLSSSINSIQEKQDVYEKESKFLESLNCQTIRFCTYTERLLLVVFSKCWKVYDYCDFSLLLTEVSRNGQFFAGGEVIAAHRILIWTEDGHSYIYQLLNSGLSKSIYPADGRVLKETIYPHLLCSTSVQENKEQSRPFVMGYMNERKEPFYKVLFSGEVSGRITLWHIPDVPVSKFDGSPREIPVTATWTLQDNFDKHDTMSQSIIDYFSGLKDGAGTAVVTSSEYIPSLDKLICGCEDGTIIITQALNAAKARLLEGGSLVKDSPPHKVLKGHHQSVTSLLYPHGLSSKLDQSWMLSGDLDSCVILWDIFTEEILHKFFLEAGPVTSLLMSPEKFKLRGEQIICCVCGDHSVALLHLEGKSCLLHARKHLFPVRMIKWHPVENFLIVGCADDSVYIWEIETGTLERHETGERARIILNCCDDSQLVKSVLPIASETLKHKSIEQRSSSPYQLGPLPCPGLQVESSCKVTDAKFCPRPFNVLPVKTKWSNVGFHILLFDLENLVELLLPTPLSDVDSSSSFYGGEVLRRAKSTVEKKTLTLRKSKTACGPLSAEALAKPITESLAQGDNTIKFSE**E**NDGIKRQ**KKMKISKKMQ**PKPSR**K**VDASLTIDTAKLFLSCLLPWGVDKDLDYLCIKHLNILKLQGPISLGISLNEDNFSLMLPGWDLCNSGMIKDYSGVNLFSRKVLDLSDKYTATLPNQVGIPRGLENNCDSLRESDTIVYLLSRLFLVNKLVNMPLELACRVGSSFRMESIHNKMRGAGNDILNMSSFYSCLRNGKNESHVPEADLSLLKLISCWRDQSVQVTEAIQAVLLAEVQQHMKSLGKI**PVN**SQPV**SM**AENGNCEMKQMLPKLEWTEELELQCVRNTLPLQ**TPVSPVKHDSNSNSANFQDVEDMPDRCALEESESPGEPRH**HSWIAKVCPCKVS	Sequence: 1102 aa Predicted IDR: 57 Percent of disordered region: 5.1% (Included)
DLX3: distal-less homeobox 3	Homeobox protein DLX-3	>NP_005211.1 **MSGSFDR**KLSSILTDISSSLS**CHAGSKDSPTLPESSVTD**LGYYSAPQHDYYS**GQ**PYGQTVNPYTYHHQFNLNGLAGTGAYSPKSEYTYGASYRQYGAY**R**EQP**LPAQDPVSVKEEP**EAEV**RMVNGKPK**KVRKPRTIYSSYQLAALQRRFQKAQYLALPERAELAAQLGLTQTQVKIWFQNRRSKFKK**LYKNGEVPLEHSPNNSDSMACNSPPSPALWDTSSHSTPAPARSQLPPPLPYSASPSYLDD**P**TNSWYHAQNLSGPHLQQQPPQPATLHHASPGPPPNPGAVY**	Sequence: 287 aa Predicted IDR: 149 Percent of disordered region: 51.9%
FAM20A: FAM20A golgi associated secretory pathway pseudokinase	Pseudokinase FAM20A isoform a precursor	>NP_060035.2 MPGLRRDRLLTLLLLGALLSADLYFHLWPQVQRQLRP**RERPRGCPCTGRASSLARDSAAA**ASD**PGTIVHNFSRTEPRTEPAGGSHSGSS**SKLQALFAHPLYNVPEEPPLLGAEDSLLASQEALRYYRRKVARWNRRHKMYREQMNLTSLDPPLQLRLEASWVQFHLGINRHGLYSRSSPVVSKLLQDMRHFPTISADYSQDEKALLGACDCTQIVKPSGVHLKLVLRFSDFGKAMFKPMRQQRDEETPVDFFYFIDFQRHNAEIAAFHLDRILDFRRVPPTVGRIVNVTKEILEVTKNEILQSVFFVSPASNVCFFAKCPYMCKTEYAVCGNPHLLEGSLSAFLPSLNLAPRLSVPNPWIRSYTLAGKEEWEVNPLYCDTVKQIYPYNNSQRLLNVIDMAIFDFLIGNMDRHHYEMFTKFGDDGFLIHLDNARGFGRHSHDEISILSPLSQCCMIKKKTLLHLQLLAQADYRLSDVMRESLLEDQLSPVLTEPHLLALDRRLQTILRTVEGCIVAHGQQSVIVDGPVEQLAP**DSGQANLTS**	Sequence: 541 aa Predicted IDR: 58 Percent of disordered region: 10.7% (Included)
ACP4: Acid phosphatase 4	Testicular acid phosphatase precursor	No agreed upon disordered regions found.	(Included)
KLK4: Kallikrein related peptidase 4	Kallikrein-4 isoform 1 preproprotein	No match	(Included)
AMELX: Amelogenin X-linked	Amelogenin, X isoform 3 precursor	>NP_001133.1 MGTWILFACLLGAAFAMPLPPHPGHPGYINFSYEVLTPLKWYQSIRPPYPSYGYEPMGGWLHHQIIPVLS**QQHPPTHTLQPHHHIPVVPAQQPVIPQQPMMPVPGQHSMTPIQHHQPNLPPPAQQPYQPQPVQPQPHQPMQPQPPVHPMQPLPPQPPLPPMFPMQPLPPMLPDLTLEAWPSTDKTKREEVD**	Sequence: 191 aa Predicted IDR: 121 Percent of disordered region: 63.3%
Sp6: Sp6 transcription factor	Transcription factor Sp6	>XP_006722178.1 MLTAVC**GSLGSQHTEAPHASPPRLDLQPLQTYQGHTSPEAGDYPSPLQPGELQSLPLGPEVDFSQGYELPGASSRVTCEDLESDSPLAPGPFS**KLLQPDMSHHYESWFRPTHPGAEDGSWWDLHPGTSWMDL**PHTQGALTSPGHPGALQAGL**GG**YVGDHQ**LCAPPP**HPHAHHLLPAAGGQHLLGPPDGAKALEVAAPESQGLDSSLDGAARPKGSRRSVPRS**SGQTVCRCPNCLEAERLGAPCGPDGGKKKHLHNCHIPGCGKAYAKTSHLKAHLRWHSGDRPFVCNWLFCGKRFTRSDELQRHLQTHTGTKKFPCAVCSRVFMRSDHLAKH**MKTHEGAKEEAAGAASGEGKAGGAVEPPGGKGKREAEGSVAPSN**	Sequence: 376 aa Predicted IDR: 213 Percent of disordered region: 56.6%

### C. Homology models of wildtype and mutant proteins

I-TASSER successfully generated 3D models for mutant proteins. Ramachandran plot and Z-score from the ProSA program indicated good quality for most wildtype and mutant proteins generated by I-TASSER. Partial structural similarity was observed between some models of the wildtype proteins generated by I-TASSER and AlphaFold (S1 Table). When I-TASSER-generated structures were compared between the wildtype and the mutants, structural changes (RMSD >1.5) were observed for several mutants (S1 Table). For mutations that cause only minor RMSD changes, the mutated site is likely critical to the protein’s function, such as substrate binding, proteolytic cleavage, or interactions with other molecules [52].

**Fig 2:**
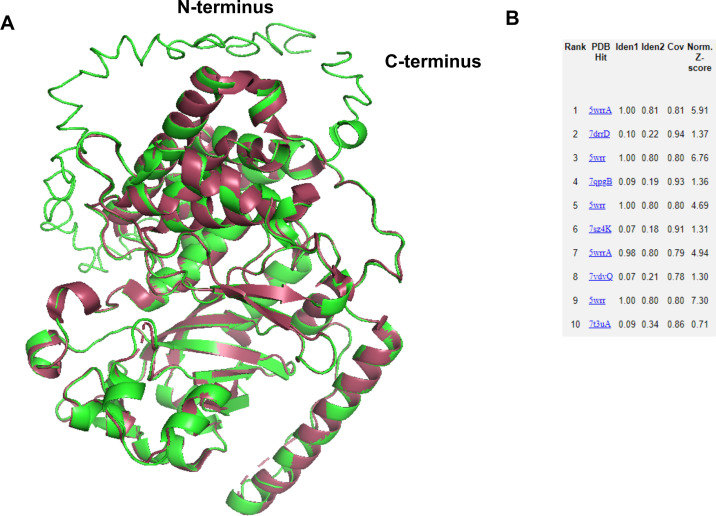
Evaluation of the homology model for accuracy. FAM20A (secretory pathway pseudokinase) has its crystal structure, 5WRR in PBD. Structure superimposition between FAM20A WT homology model and 5WRR showed good alignment between the two structures, except the N-terminal that was missing in 5WRR. The RMSD value for the alignment was 0.291 (2522 to 2522 atoms) (A). I-TASSER output for FAM20A homology model, showing 5WRR is used as template for modeling the FAM20A amino acid sequence (B).

To further evaluate the accuracy of homology models developed using I-TASSER, the modeled structure of FAM20A was compared with its closely related crystal structure available in PDB. Structure superimposition between the homology model of FAM20A and its partial crystal structure 5WRR from PBD showed good backbone alignment with a RMSD value of 0.291 (2522–2522 atoms), except for the N-terminal that was missing in 5WRR (Fig 2A). An RMSD value below 2 Å is considered a good alignment between two structures. I-TASSER is programmed to identify best-match templates from PDB using a meta-server threading approach. The output of I-TASSER showed that 5WRR was identified as a template for modeling the FAM20A sequence (Fig 2B). As PDB does not have closely related crystal structures for the other proteins included in our list, this step was performed only for FAM20A.

## Discussion

The lack of experimentally determined structures is one bottleneck in research focusing on proteins involved in dental anomalies and tooth development. *In silico* protein structure prediction depends on two facts: (i) the protein 3D structure is determined by its amino acid sequence, and (ii) the change in protein structure happens at a slower rate compared to the sequence during evolution [53]. In this study, I-TASSER was used to generate complete models for the wildtype and mutant proteins. Besides template dependence, I-TASSER also applies *ab initio* structure modeling for protein targets in the regions with weak homologous (<30% identity) templates, allowing us to generate a complete model for mutant proteins with sequence variation from their wildtype partners [26].

For a given protein sequence input, I-TASSER predicts the 3D structure and provides results along with key metrics such as C-scores, TM-scores, and RMSD values. The C-score reflects the quality of the predicted model and is derived from the significance of threading template alignments as well as the convergence of structure assembly simulations. It typically ranges from -5 to 2, with a higher C-score indicating a model with greater confidence and reliability [26].

RMSD (Root Mean Square Deviation) and TM-score (Template Modeling Score) are used to assess the similarity between protein structures, differing in their approach and sensitivity. RMSD measures the average distance between corresponding atoms. For proteins, like in our study, where the reliable template is not always available, I-TASSER calculates the RMSD of the predicted models relative to the native structures based on the C-score [26,53,54]. TM-score focuses on topological similarity. Protein pairs with a TM-score >0.5 are mostly in the same fold, while those with a TM-score <0.5 are mainly not in the same fold [54].

It is essential to note that the C-score and prediction confidence can vary along the length of the protein, particularly if the protein contains IDRs. To improve the reliability of the predicted protein, we screened the protein sequences for IDRs before using them as input in I-TASSER. Of the proteins we included in our study, 18% of the ODAPH sequence was identified as intrinsically disordered (Table 3), resulting in a homology model of the wildtype protein with the least reliable matrix (C-score = −4.30, TM-score = 0.26 ± 0.08, RMSD = 14.6 ± 3.7Å) (S1 Table)

Besides evaluating I-TASSER-generated models using a Ramachandran plot, RMSD, TM, and Z-scores, the wild-type models were also compared with those from AlphaFold. Similarity in the folding pattern was observed between the I-TASSER and AlphaFold models for most proteins, except for ODAPH, which is attributed to a lack of a faithful template for this protein (S1 Table). Furthermore, we identified 18% of the ODAPH sequence as intrinsically disordered (Table 3), resulting in a model with a relatively low C-score in I-TASSER.

Truncation mutations are the most frequently identified mutations associated with AI. However, many truncation mutations, along with certain frameshift mutations, are subject to NMD, which prevents the expression of the corresponding protein. As a result, our study excluded truncation mutations from further analysis and concentrated on missense and selected frameshift mutations. To identify possible structural changes in protein variants, the I-TASSER-generated wildtype and mutant protein models were superimposed in PyMOL. Considering sequence variability caused by several frame-shift mutations, a sequence-independent algorithm (Super) was employed for structure comparison, which utilizes a dynamic programming approach to identify the optimal structural match. The alignment RMSD of 1.5 or higher was identified as indicating structural changes between the wild-type and mutant proteins (S1 Table). Several of the structural variations observed in our study are found to be supported by experimental evidence. Wang et al., 2014 identified a double substitution mutation in ITGB6 in a patient with AI [55]. Our modeling showed no structural change in A143T and H275Q variant alone in ITGB6 (S1 Table); however, the double mutation altered a portion of the protein located away from the mutation site. A partial crystal structure of Integrin beta-6 (chains B, D in 4um8) is available in the Protein Data Bank. A structural comparison between the PDB, AlphaFold and I-TASSER model of ITGB6 showed modeling resemblance over the core part of the protein, containing the double mutation. Considering the location of the structural alteration in the ‘twilight zone,’ it is considered less reliable, and the functional effect of the double mutant is likely related to the pathogenic impact of the conserved function of A143 and H275. H275 in exon 6 is conserved among its vertebral orthologs mediating subunit interaction [55]. A143 in exon 4 is another evolutionary conserved amino acid located within a specific metal ion-dependent adhesion site. Mutations in H275 and A143 would likely prevent the subunit interaction and binding to extracellular matrix respectively, resulting in a loss of function [55].

Glutamic acid (E) at a well-conserved position of 133 in ACP4 is involved in homodimerization. Substitution of a negatively charged amino acid with a positively charged one (E133K) has shown minor structural alteration in our study (S1 Table). Aligned with our finding, mutations in this conserved region are suggested to alter size and charge, thus interfering with homodimerization [47]. Most other missense mutations in our list are in conserved regions and are likely to affect protein function without causing major structural changes. For example, two substitution mutations in H226 and H204 regions of MMP20 did not have an apparent structural change. It is thus unclear if these mutations can affect zinc-binding, crucial to MMP20 function [56].

With the completion of the human genome project and ongoing advances in sequencing technologies, millions of genetic variants have been discovered, many of which remain poorly understood. Computational protein modeling presents a powerful approach to help bridge this knowledge gap by facilitating the interpretation of clinically relevant, potentially pathogenic variants [57]. These models not only enable the study of previously uncharacterized proteins but also provide rapid insights crucial for addressing time-sensitive clinical scenarios [57].

We acknowledge several limitations of our study. First, the use of a single protein modeling tool, I-TASSER, may limit the generalizability of our findings. Additionally, the number of proteins and variants analyzed was constrained by the presence of IDRs in the protein sequences. Despite these limitations, our study highlights the potential of using computational models to investigate protein variants.It is essential to be aware that the modeller generated the RMDS and other scores for the entire protein sequence, which may not be accurate for the region of interest. A careful consideration of models, templates, alignment over the regions of interest and supporting literature is necessary to predict the possible structural impact of a variant causing human diseases like Amelogenesis Imperfecta.

## Supporting information

S1 Table Homology model of the proteins and mutants involved in Amelogenesis Imperfecta. (PDF)
