# Supplementary material for: Using computer-generated protein models to analyze mutations linked to Amelogenesis Imperfecta
Source: PLoS One. 2025 Jun 26;20(6):e0326679. doi: 10.1371/journal.pone.0326679 (PMC12200857; doi:10.1371/journal.pone.0326679)
Supplement: S1 Table — (PDF) [file pone.0326679.s001.pdf]

S1 Table. Homology model of the proteins and mutants involved in Amelogenesis Imperfecta

**LAMB3: Laminin subunit beta 3**		
LAMB3 WT<br>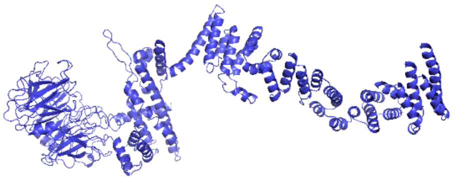<br>Electrostatic Potential: LAMB3 WT<br>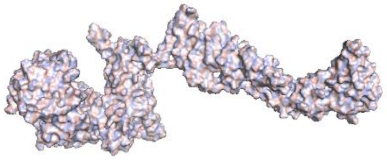	C-score=0.17<br>TM-score = 0.74±0.11<br>RMSD = 8.9±4.6Å<br><br>Z-Score: -5.47<br>Ramachandran plot<br>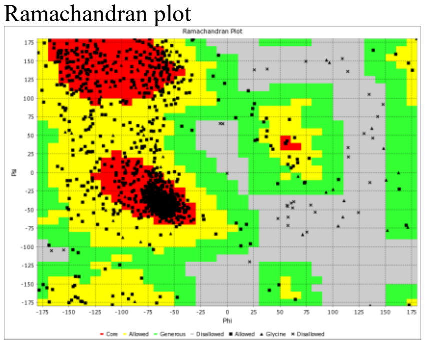	AlphaFold model of the WT protein<br>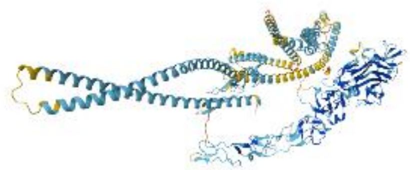
Truncation: S1144X<br>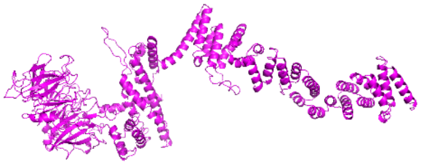<br>Electrostatic Potential: S1144X<br>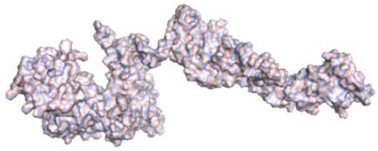	C-score=0.04<br>TM-score = 0.72±0.11<br>RMSD = 9.1±4.6Å<br><br>Z-Score: -5.93<br>Ramachandran plot<br>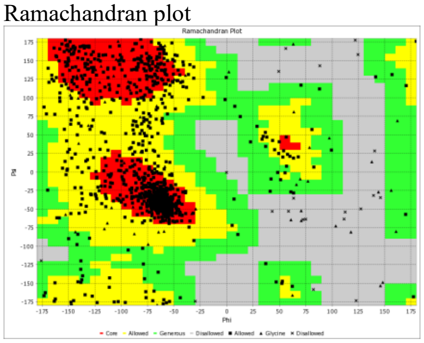	Structure superimposition: LAMB3 WT & S1144X<br>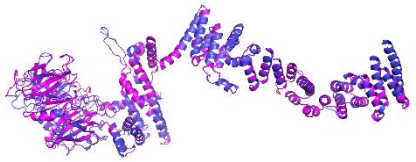<br>RMSD = 0.766 (9749 atoms)
Frameshift: 8-BP DEL, NT3446<br>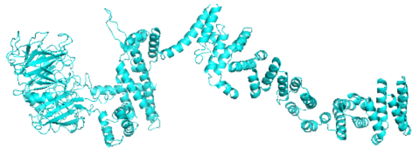<br>Electrostatic Potential: 8-BP DEL, NT3446<br>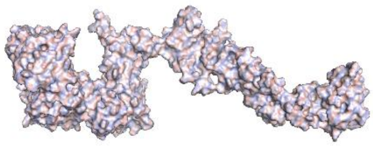	C-score=-0.08<br>TM-score = 0.70±0.12<br>RMSD = 9.4±4.6Å<br><br>Z-Score: -5.33<br>Ramachandran plot<br>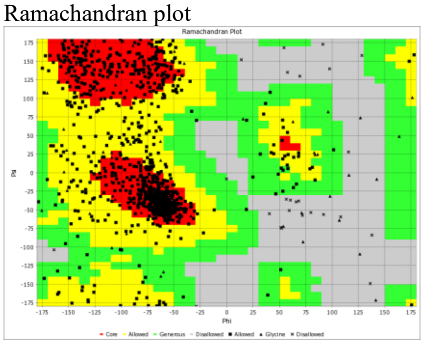	Structure superimposition: LAMB3 WT & 8-BP DEL, NT3446<br>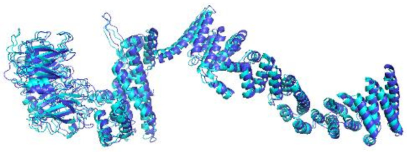<br>RMSD = 0.749 (8390 atoms)

Frameshift: 1-BP INS, 3392G<br>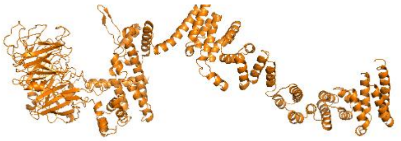<br>Electrostatic Potential: 1-BP INS, 3392G<br>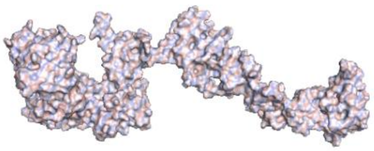	C-score=0.04<br>TM-score = 0.72±0.11<br>RMSD = 9.1±4.6Å<br><br>Z-Score: -5.28<br>Ramachandran plot<br>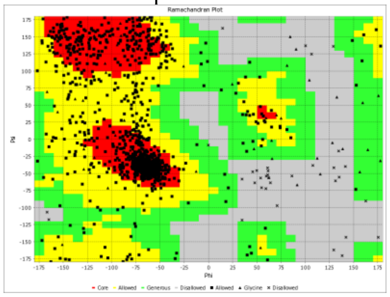	Structure superimposition: LAMB3 WT & 1-BP INS, 3392G<br>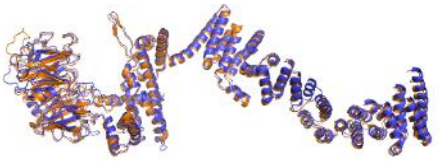<br>RMSD = 0.908 (8270 atoms)
**ITGB6: integrin subunit beta 6**		
ITGB6 WT<br>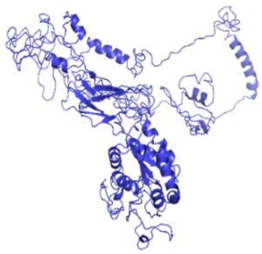<br>Electrostatic Potential: ITGB6 WT<br>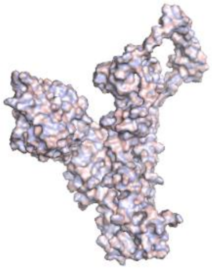	C-score: -1.21<br>TM-score: 0.56±0.15<br>RMSD = 11.3±4.6Å<br><br>Z-Score: -7.02<br>Ramachandran plot<br>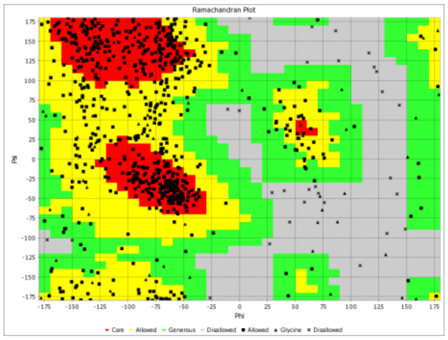	AlphaFold model of the WT protein<br>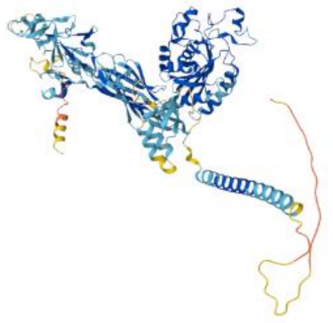
Substitution: A143T<br>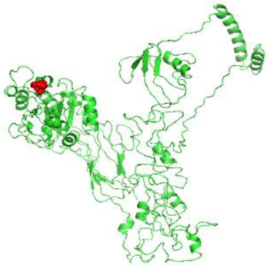<br>Electrostatic Potential: A143T<br>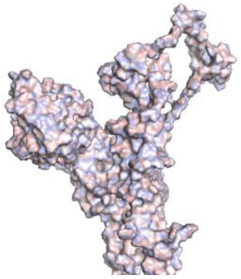	C-score: -1.25<br>TM-score: 0.56±0.15<br>RMSD = 11.4±4.5Å<br><br>Z-Score: -7.28<br>Ramachandran plot<br>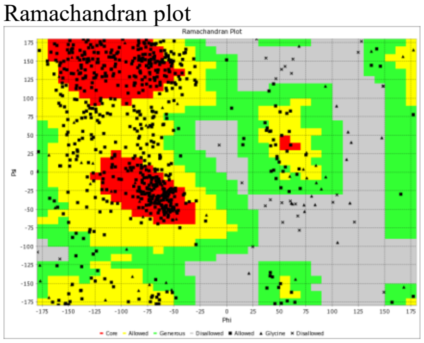	Structure superimposition: ITGB6 WT and A143T<br>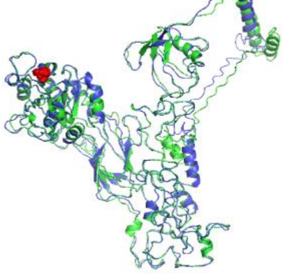<br>RMSD = 1.736 (8794 atoms)

Substitution: H275Q<br>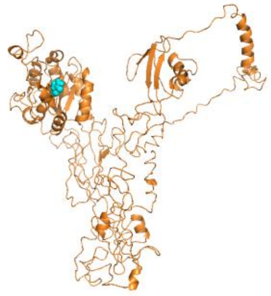<br>Electrostatic Potential: H275Q<br>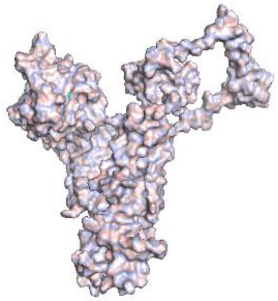	C-score: -1.09<br>TM-score: 0.58±0.14<br>RMSD = 11.0±4.6Å<br><br>Z-Score: -7.28<br>Ramachandran plot<br>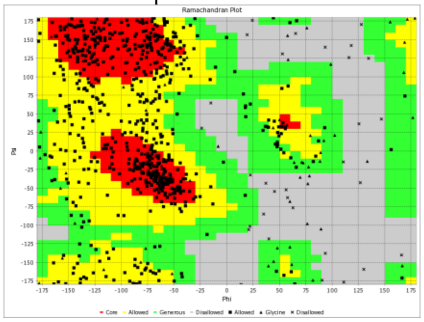	Structure superimposition: ITGB6 WT & H275Q*<br>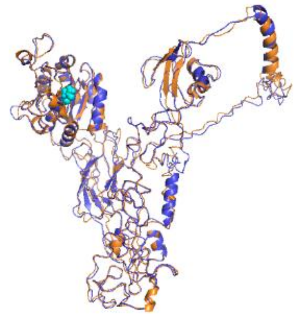<br>**RMSD = 1.744 (8367 atoms)**
Double Mutation: A143T+ H275Q<br>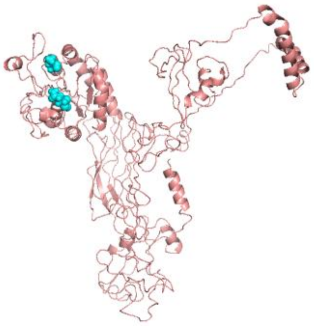<br>Electrostatic Potential: A143T+ H275Q (Double mutant)<br>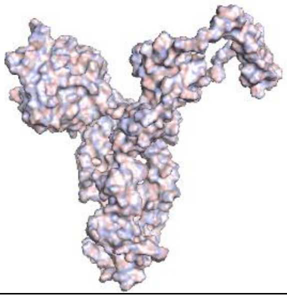	C-score=-0.95<br>TM-score = 0.59±0.14<br>RMSD = 10.6±4.6<br><br>Z-Score: -7.06<br>Ramachandran plot<br>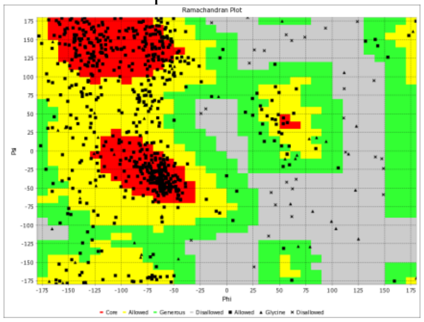	Structure superimposition: ITGB6 WT & A143T+ H275Q (Double mutant) *<br>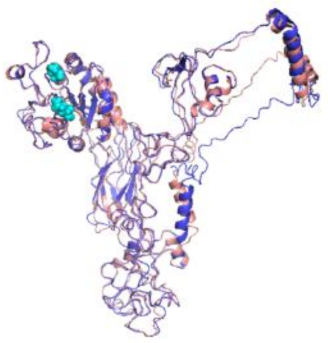<br>**RMSD = 1.497 (7880 atoms)**

<table>
<tr>
<td>Substitution: P196T
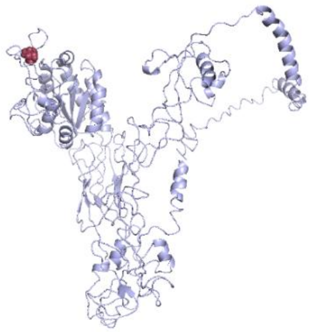
Electrostatic Potential: P196T
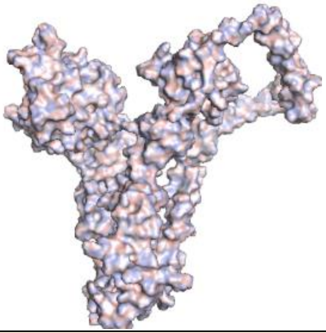</td>
<td>C-score=-1.18
TM-score = 0.57±0.15
RMSD = 11.2±4.6

Z-Score: -7.12
Ramachandran plot
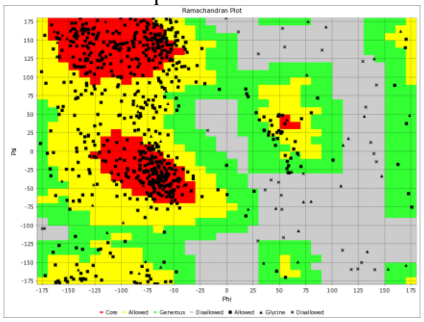
</td>
<td>Structure superimposition: ITGB6 WT & P196T
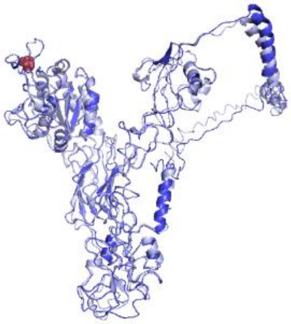
RMSD = 1.385 (7322 atoms)</td>
</tr>
<tr>
<td colspan="3">ODAPH: Odontogenesis Associated Phosphoprotein</td>
</tr>
<tr>
<td>ODAPH WT
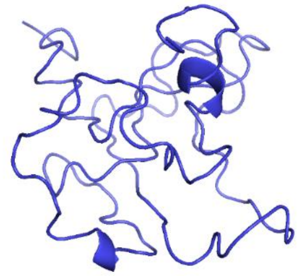
Electrostatic Potential: ODAPH WT
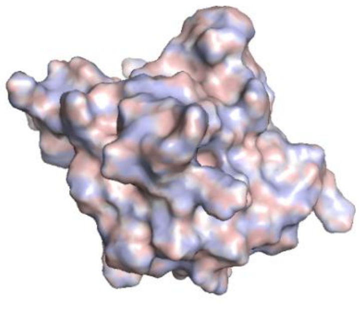</td>
<td>C-score=-4.30
TM-score = 0.26±0.08
RMSD = 14.6±3.7Å

Z-Score: -3.46
Ramachandran plot
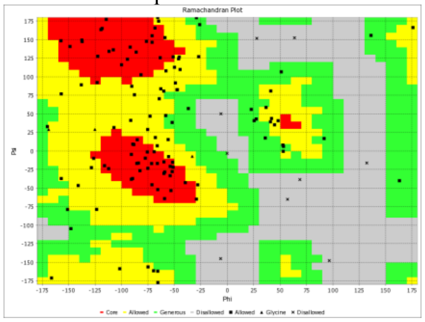
</td>
<td>AlphaFold model of the WT protein
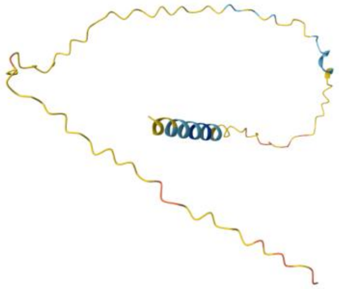</td>
</tr>
</table>

In-frame deletion/insertion:<br>6-BP DEL/16-BP INS<br>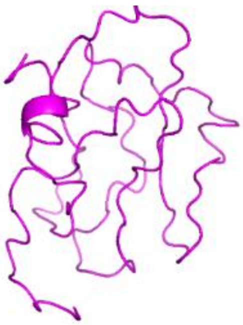<br>Electrostatic Potential: 6-BP DEL/16-BP INS<br>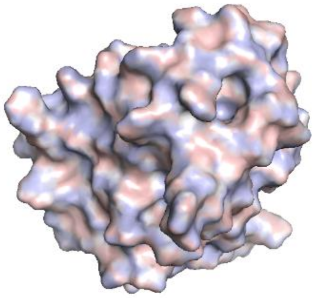	C-score=-4.81<br>TM-score = 0.22±0.06<br>RMSD = 16.1±3.1Å<br><br>Z-Score: -4.75<br>Ramachandran plot<br>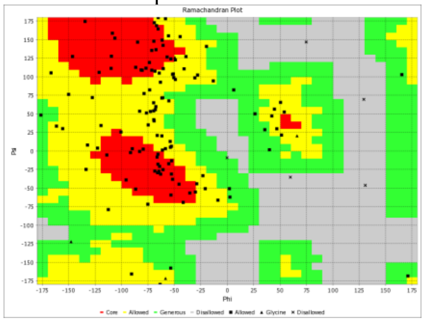	Structure superimposition: ODAPH WT & BP INS*<br>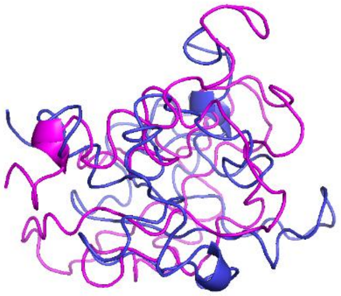<br>**RMSD = 2.635 (89 atoms)**
**SLC10A7: Solute carrier family 10 member 7**		
SLC10A7 WT<br>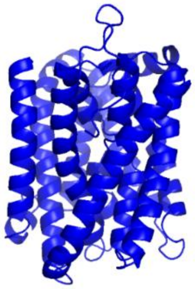<br>Electrostatic Potential: SLC10A7 WT<br>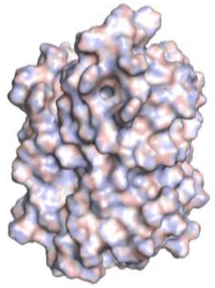	C-score=0.97<br>TM-score = 0.85±0.08<br>RMSD = 4.4±2.9Å<br>*Z*-Score: -4.52<br><br>Ramachandran plot<br>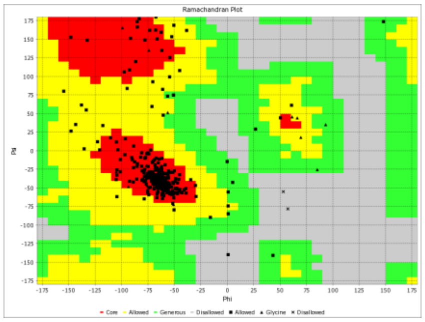	AlphaFold model of the WT protein<br>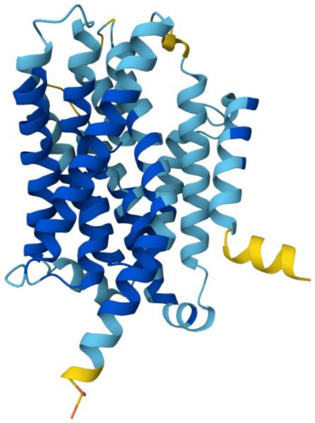

<table>
<tr>
<td>Substitution: L74P
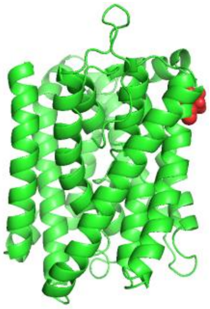
Electrostatic Potential: L74P
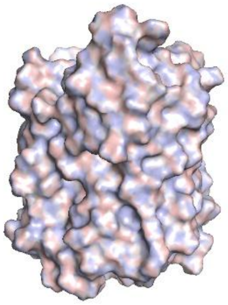</td>
<td>C-score=0.96
TM-score = 0.84±0.08
RMSD = 4.5±3.0Å
Z-Score: -4.36

Ramachandran plot
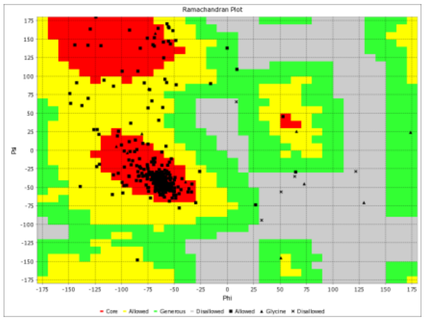</td>
<td>Structure superimposition: SLC10A7 WT & L74P
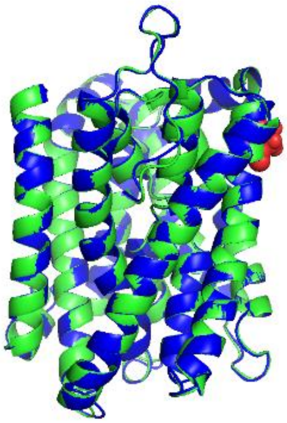
RMSD = 0.544 (3491 atoms)</td>
</tr>
<tr>
<td>Substitution: G130R
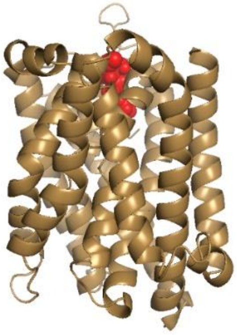
Electrostatic Potential: G130R
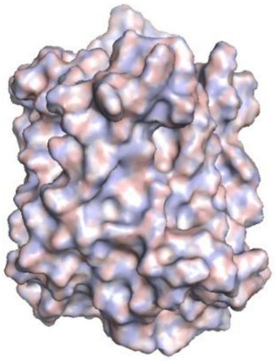</td>
<td>C-score=0.98
TM-score = 0.85±0.08
RMSD = 4.4±2.9Å

Z-Score: -4.4
Ramachandran plot
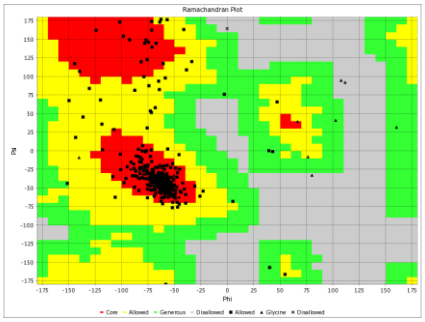</td>
<td>Structure superimposition: SLC10A7 WT & G130R
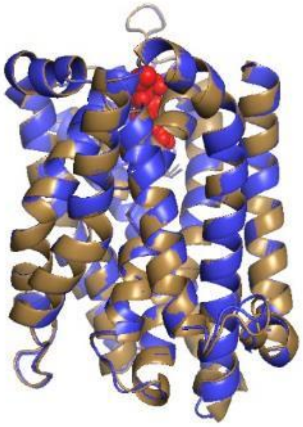
RMSD = 0.632 (3531 atoms)</td>
</tr>
</table>

**MMP20: Matrix metallopeptidase 20**		
MMP20 WT 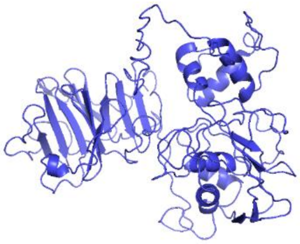 Electrostatic Potential: MMP20 WT 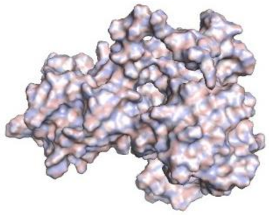	C-score=0.00<br>TM-score = 0.71±0.11<br>RMSD = 7.2±4.2Å<br><br>*Z*-Score: -8.16<br>Ramachandran plot 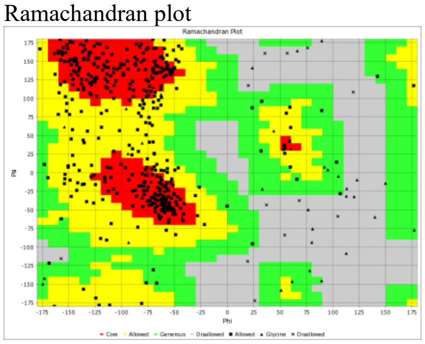	AlphaFold model of the WT protein 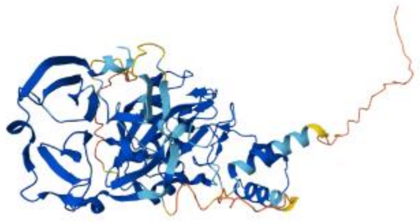
Substitution: H226Q 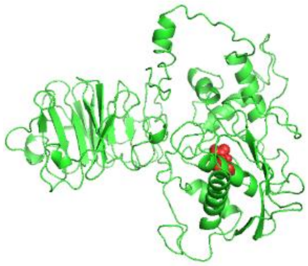 Electrostatic Potential: H226Q 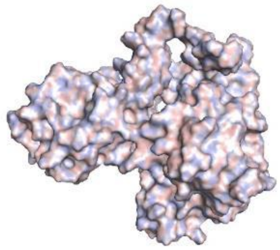	C-score=0.12<br>TM-score = 0.73±0.11<br>RMSD = 7.0±4.1Å<br><br>*Z*-Score: -8.41<br>Ramachandran plot 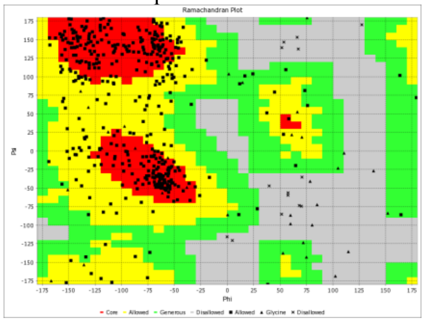	Structure superimposition: MMP20 WT & H226Q 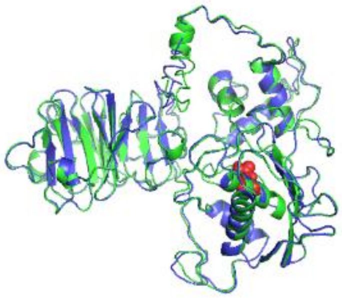 RMSD = 0.623 (4708 atoms)
Substitution: H204R 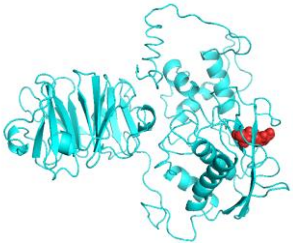 Electrostatic Potential: H204R 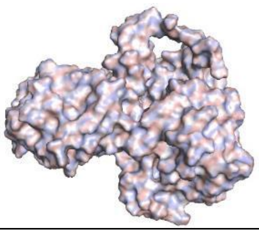	C-score=0.03<br>TM-score = 0.72±0.11<br>RMSD = 7.2±4.2Å<br><br>Z-Score: -7.85<br>Ramachandran plot 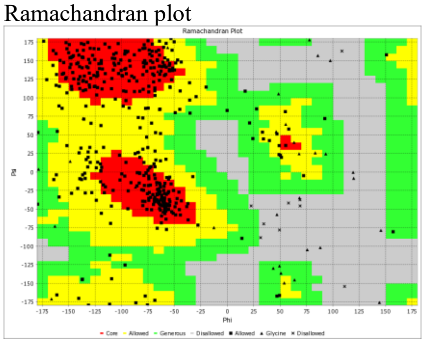	Structure superimposition: MMP20 WT & H204R 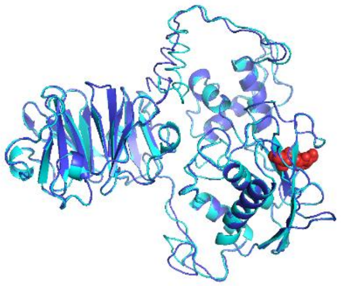 RMSD = 0.538 (4724 atoms)

GPR68: G protein-coupled receptor 68		
GPR68 WT<br>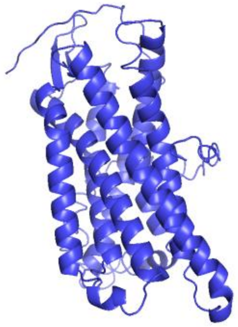<br>Electrostatic Potential: GPR68 WT<br>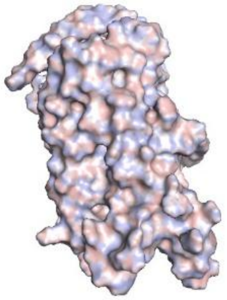	C-score=-1.54<br>TM-score = 0.53±0.15<br>RMSD = 10.2±4.6Å<br><br>*Z*-Score: -2.39<br>Ramachandran plot<br>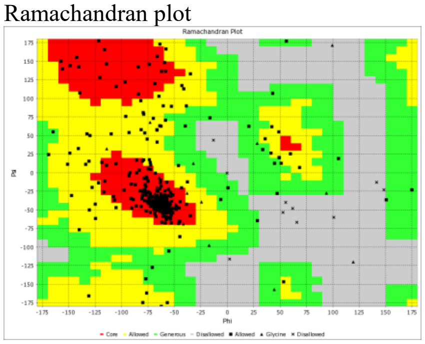	AlphaFold model of the WT protein<br>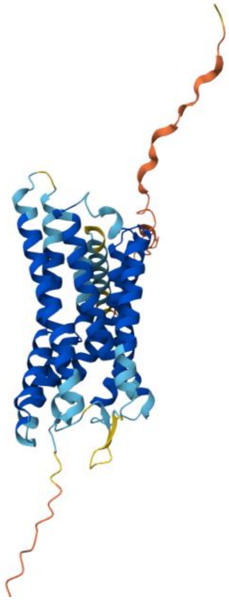
Substitution: L74P<br>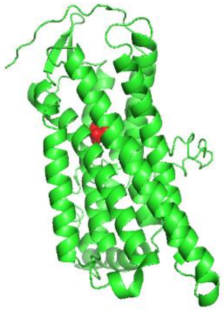<br>Electrostatic Potential: L74P<br>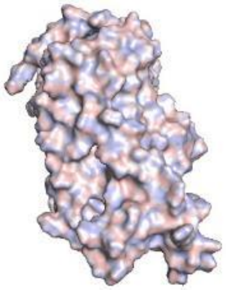	C-score=-1.69<br>TM-score = 0.51±0.15<br>RMSD = 10.5±4.6Å<br><br>Z-Score: -1.69<br>Ramachandran plot<br>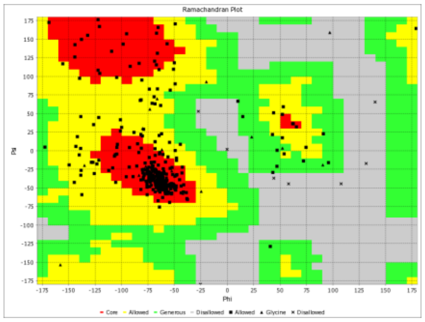	Structure superimposition: GPR68 WT & L74P*<br>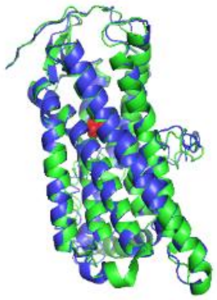<br>**RMSD = 1.503 (3840 atoms)**

In-frame deletion: 450-BP DEL, NT386<br>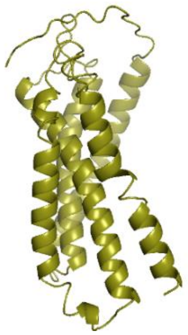<br>Electrostatic Potential: 450-BP DEL, NT386<br>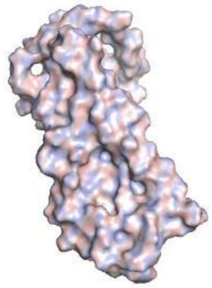	C-score=-0.21<br>TM-score = 0.69±0.12<br>RMSD = 5.9±3.7Å<br><br>*Z*-Score: -0.29<br>Ramachandran plot<br>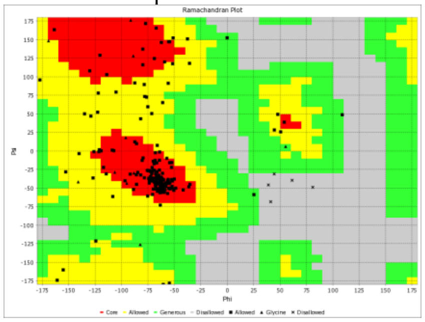	Structure superimposition: GPR68 WT & 450-BP DEL, NT386*<br>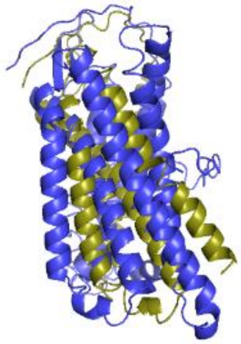<br>**RMSD = 2.234 (2273 atoms)**
Frameshift: 2-BP DEL, NT667<br>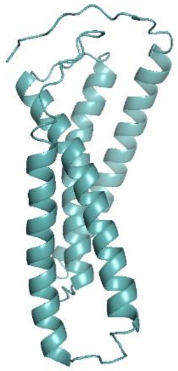<br>Electrostatic Potential: 2-BP DEL, NT667<br>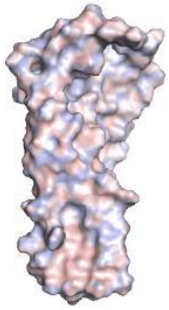	C-score=-0.37<br>TM-score = 0.67±0.13<br>RMSD = 5.8±3.6Å<br><br>*Z*-Score: -1.15<br>Ramachandran plot<br>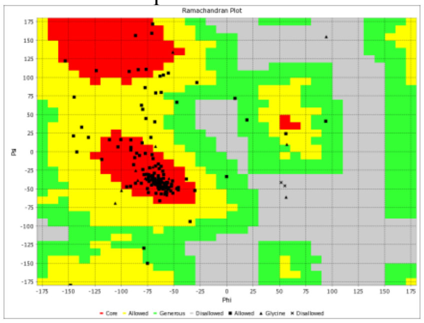	Structure superimposition: GPR68 WT & 2-BP DEL, NT667*<br>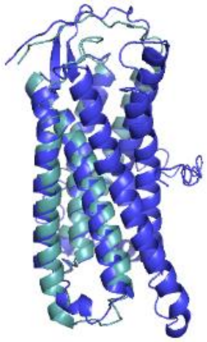<br>**RMSD = 3.618 (1640 atoms)**

<table>
<tr><th colspan="3">SLC24A4: Solute carrier family 24 member 4</th></tr>
<tr>
<td>SLC24A4 WT
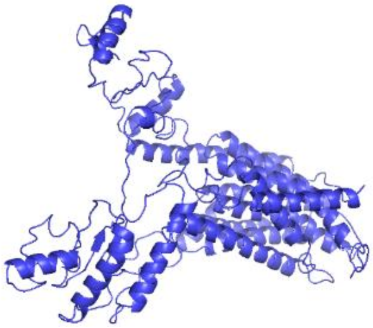
Electrostatic Potential: SLC24A4 WT
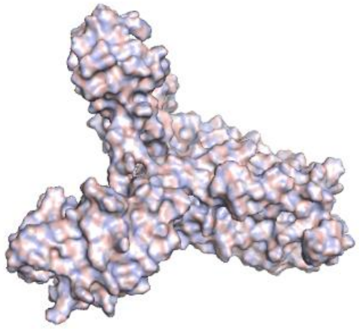</td>
<td>C-score=-0.90
TM-score = 0.60±0.14
RMSD = 9.9±4.6Å
Z-Score: -2.94

Ramachandran plot
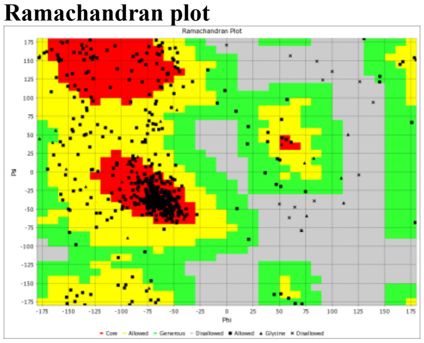
</td>
<td>AlphaFold model of the WT protein
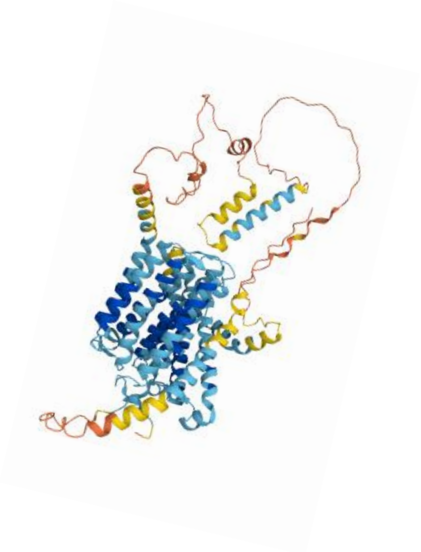</td>
</tr>
<tr>
<td>Substitution: A146V
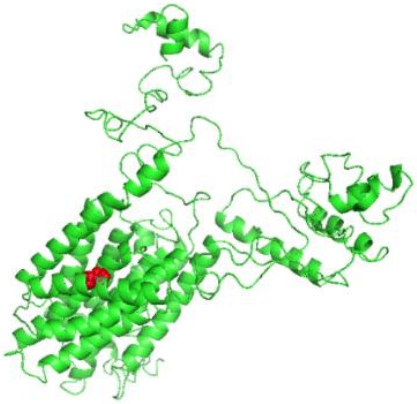
Electrostatic Potential: A146V
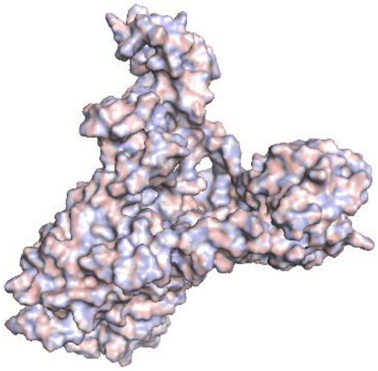</td>
<td>C-score=-0.94
TM-score = 0.60±0.14
RMSD = 10.0±4.6Å

Z-Score: -2.43
Ramachandran plot
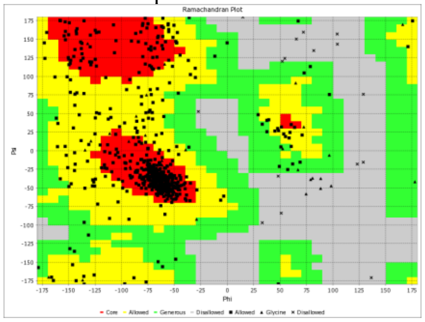
</td>
<td>Structure superimposition: SLC24A4 WT & A146V
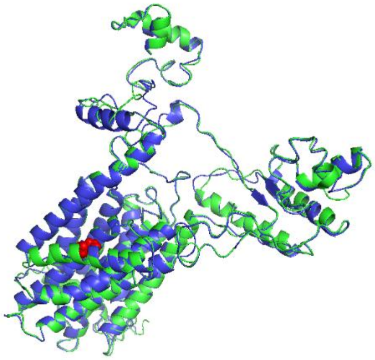
RMSD = 1.232 (5904 atoms)</td>
</tr>
</table>

Substitution: S499C<br>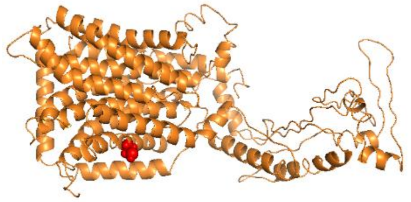<br>Electrostatic Potential: S499C<br>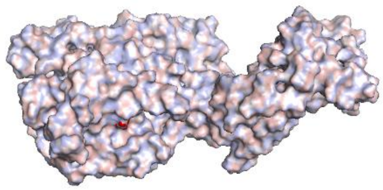	C-score=-1.17<br>TM-score = 0.57±0.15<br>RMSD = 10.6±4.6Å<br><br>Z-Score: -1.31<br>Ramachandran plot<br>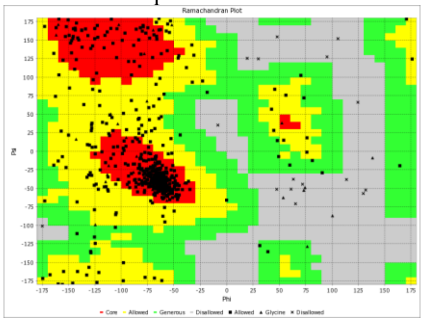	Structure superimposition: SLC24A4 WT & S499C*<br>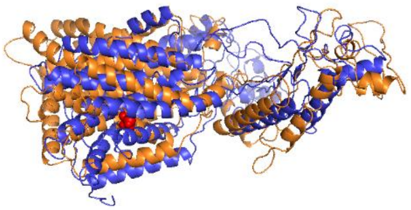<br>**RMSD = 5.693 (4175 atoms)**
**WDR72: WD repeat domain 72**		
WDR72 WT<br>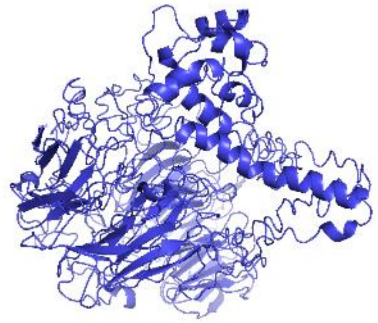<br>Electrostatic Potential: WDR72 WT<br>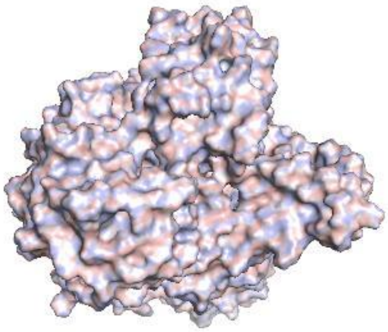	C-score=-2.60<br>TM-score = 0.41±0.14<br>RMSD = 15.9±3.2Å<br><br>*Z*-Score: -0.77<br>Ramachandran plot<br>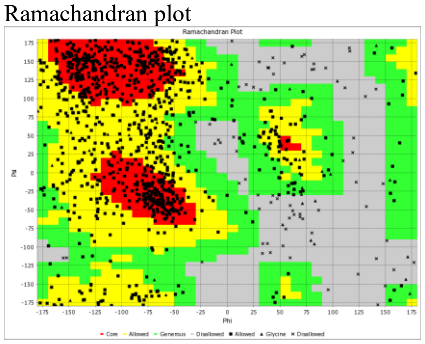	AlphaFold model of the WT protein<br>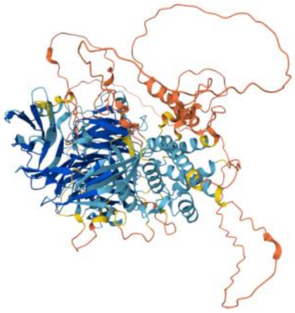

Frameshift: 2-BP DEL, 1467AT

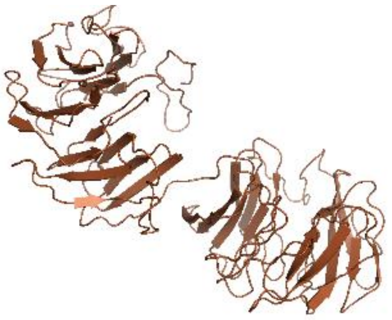

Electrostatic Potential: 2-BP DEL, 1467AT

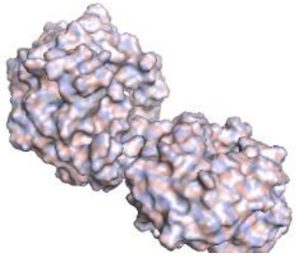

C-score=-0.86
TM-score = 0.61±0.14
RMSD = 9.3±4.6Å

Z-Score: Unable to calculate

Ramachandran plot

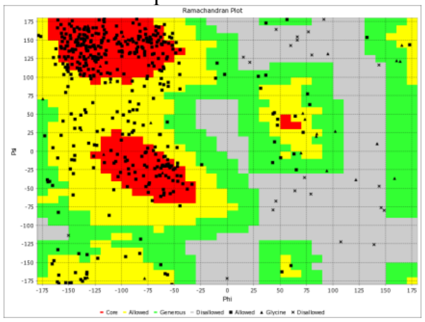

Structure superimposition: WDR72 WT & 2-BP DEL, 1467AT*

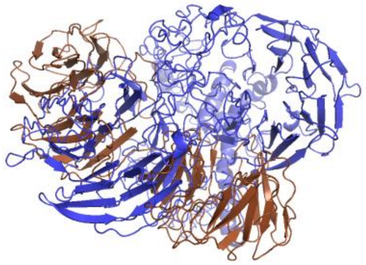

RMSD = 22.656 (4414 atoms)

FAM20A: FAM20A golgi associated secretory pathway pseudokinase

FAM20A WT

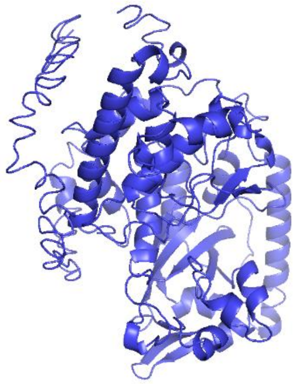

Electrostatic Potential: FAM20A WT

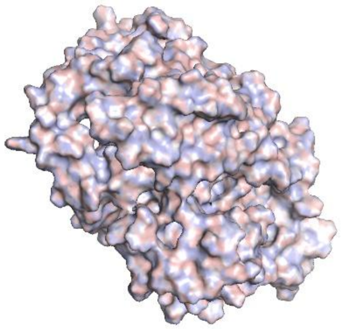

C-score=-0.55
TM-score = 0.64±0.13
RMSD = 8.7±4.6Å

Z-Score: -6.61
Ramachandran plot

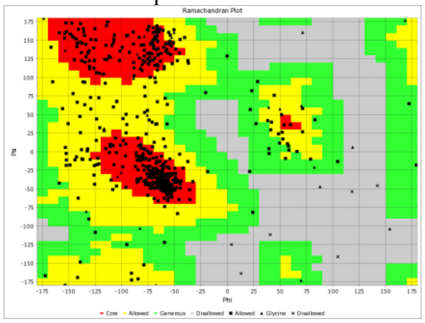

AlphaFold model of the WT protein

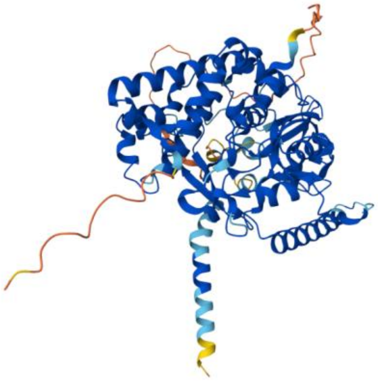

<table>
<tr>
<td>Frameshift: 2-BP DEL, 34CT
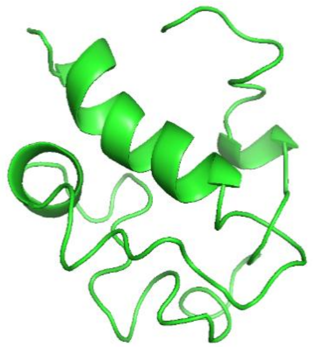
Electrostatic Potential: 2-BP DEL, 34CT
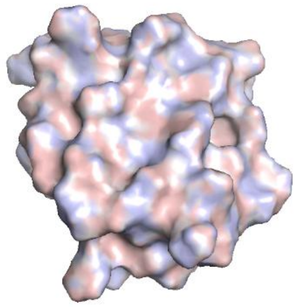</td>
<td>C-score=-3.94
TM-score = 0.29±0.09
RMSD = 12.3±4.4Å

Z-Score: not calculated.
Ramachandran plot
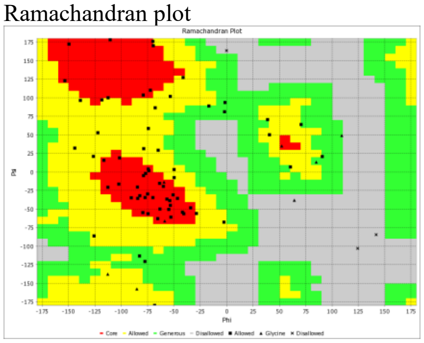</td>
<td>Structure superimposition: FAM20A WT & 2-BP DEL, 34CT*
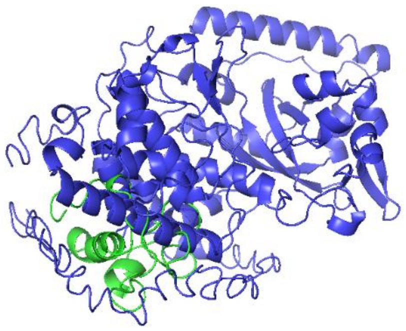
RMSD = 8.028 (262 atoms)</td>
</tr>
<tr>
<td>Frameshift: 5-BP DEL, NT1175
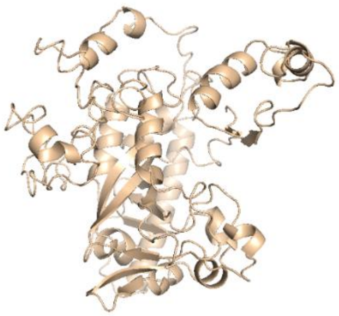
Electrostatic Potential: 5-BP DEL, NT1175
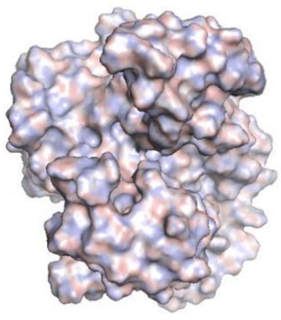</td>
<td>C-score=-0.19
TM-score = 0.69±0.12
RMSD = 7.3±4.2Å

Z-Score: -5.48

Ramachandran plot
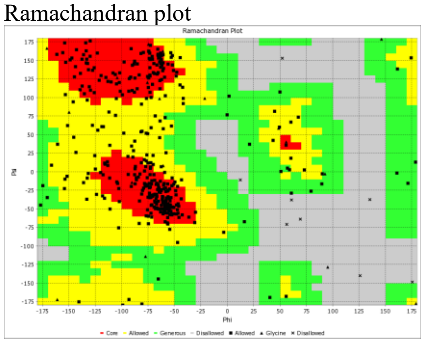</td>
<td>Structure superimposition: FAM20A WT & 5-BP DEL, NT1175
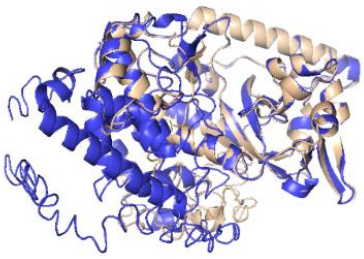
RMSD = 0.516 (3401 atoms)</td>
</tr>
</table>

<table>
<tr><th colspan="3">ACP4: Acid phosphatase 4</th></tr>
<tr>
<td>ACP4 WT
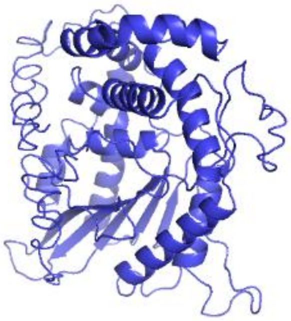
Electrostatic Potential: ACP4 WT
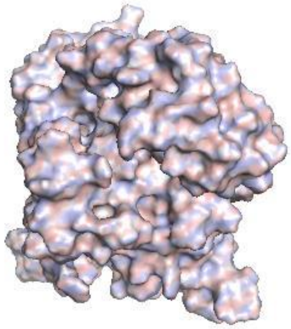</td>
<td>C-score=-1.37
TM-score = 0.55±0.15
RMSD = 10.1±4.6Å

Z-Score: -6.91
Ramachandran plot
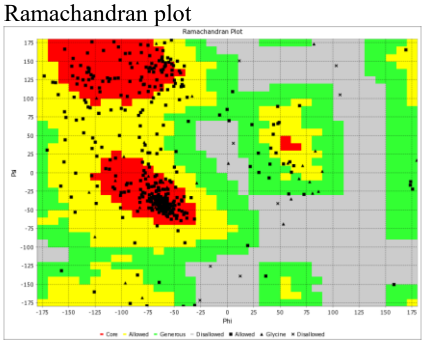</td>
<td>AlphaFold model of the WT protein
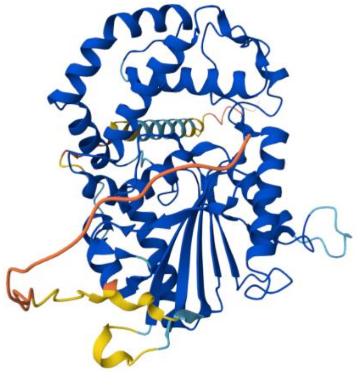</td>
</tr>
<tr>
<td>Substitution: S238L
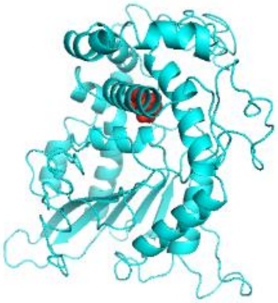
Electrostatic Potential: S238L
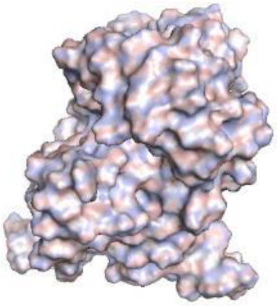</td>
<td>C-score=-1.32
TM-score = 0.55±0.15
RMSD = 10.0±4.6Å

Z-Score: -7.8
Ramachandran plot
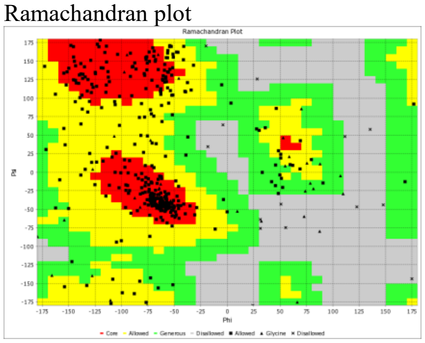</td>
<td>Structure superimposition: ACP4 WT & S238L
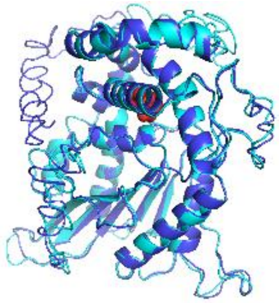
RMSD = 0.744 (3798 atoms)</td>
</tr>
</table>

Substitution: R111C<br>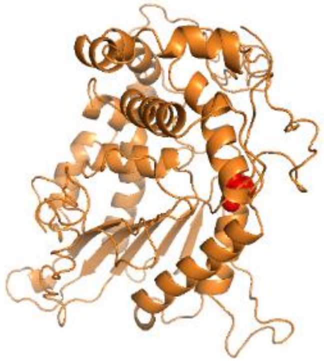<br>Electrostatic Potential: R111C<br>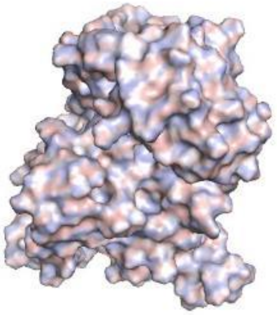	C-score=-1.41<br>TM-score = 0.54±0.15<br>RMSD = 10.2±4.6Å<br><br>Z-Score: -7.99<br>Ramachandran plot<br>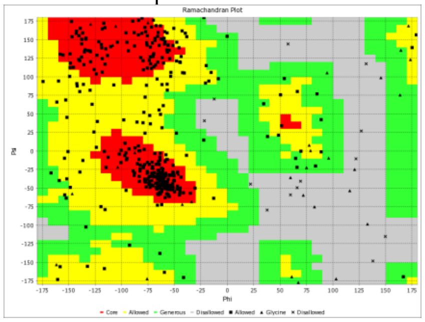	Structure superimposition: ACP4 WT & R111C<br>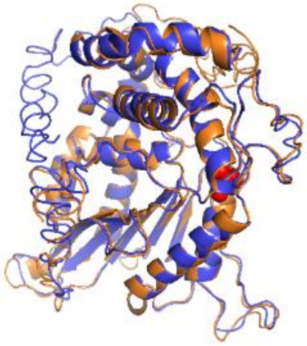<br>RMSD = 0.851 (3883 atoms)
Substitution: R76C<br>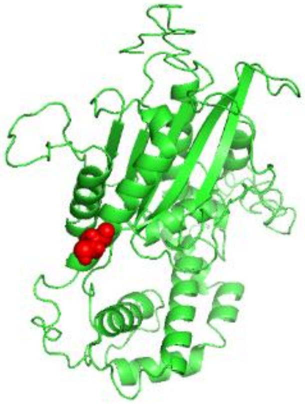<br>Electrostatic Potential: R76C<br>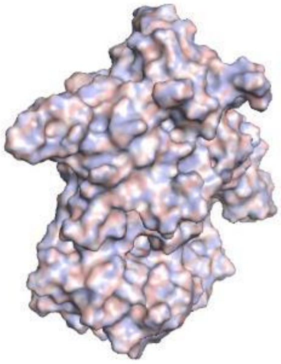	C-score=-1.72<br>Estimated TM-score = 0.51±0.15<br>Estimated RMSD = 11.0±4.6Å<br><br>Z-Score: -7.24<br>Ramachandran plot<br>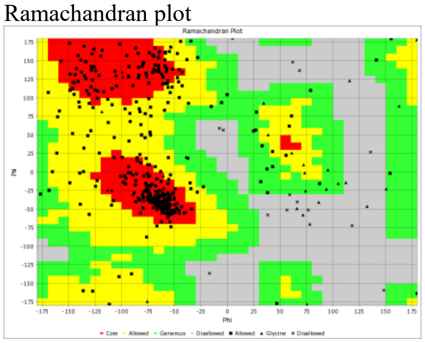	Structure superimposition: ACP4 WT & R76C<br>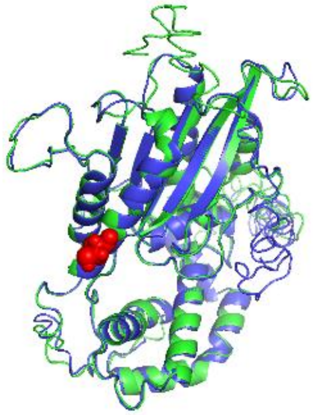<br>RMSD = 0.657 (3525 atoms)

Substitution: A128P<br>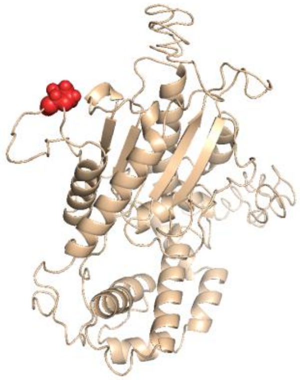<br>Electrostatic Potential: A128P<br>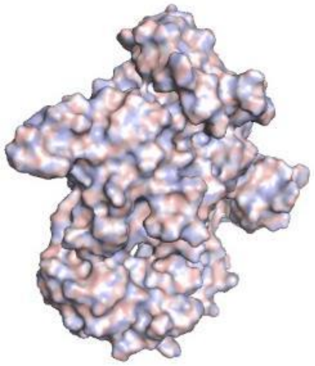	C-score=-1.74<br>TM-score = 0.50±0.15<br>RMSD = 11.0±4.6Å<br><br>Z-Score: -7.07<br>Ramachandran plot<br>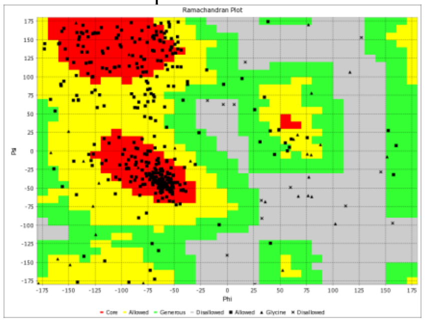 	Structure superimposition: ACP4 WT & A128P<br>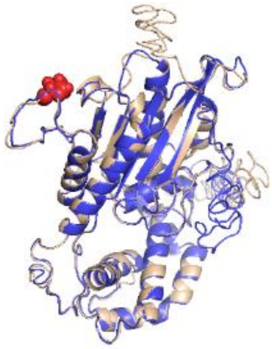<br>RMSD = 0.688 (3561 atoms)
Substitution: E133K<br>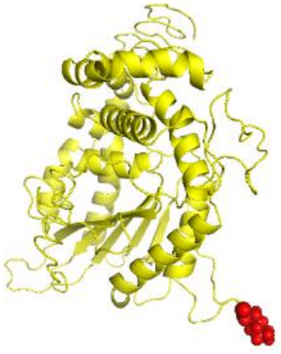<br>Electrostatic Potential: E133K<br>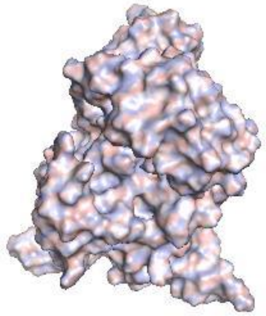	C-score=-1.62<br>TM-score = 0.52±0.15<br>RMSD = 10.7±4.6Å<br><br>*Z*-Score: -8<br>Ramachandran plot<br>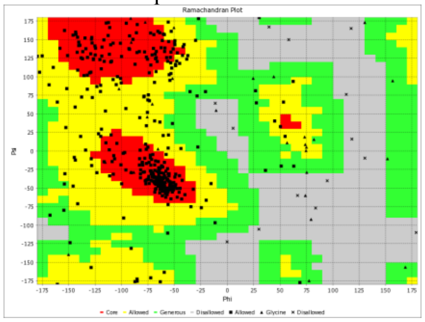 	Structure superimposition: ACP4 WT & E133K<br>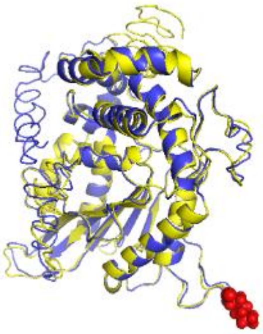<br>RMSD = 0.715 (3817 atoms)

<table>
<tr><th colspan="3">KLK4: Kallikrein related peptidase 4</th></tr>
<tr>
<td>KLK4 WT
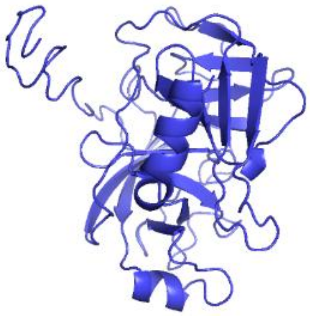
Electrostatic Potential: KLK4 WT
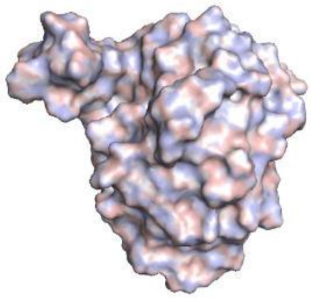</td>
<td>C-score=-0.80
TM-score = 0.61±0.14
RMSD = 7.6±4.3Å

Z-Score: -7.56
Ramachandran plot
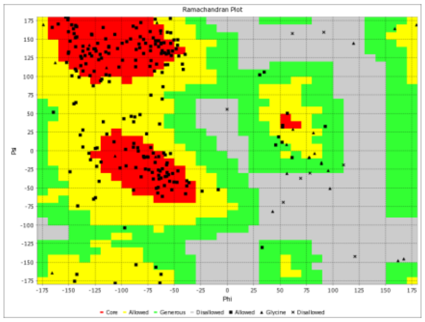</td>
<td>AlphaFold model of the WT protein
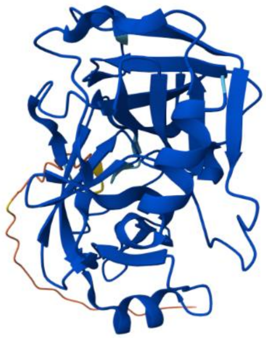</td>
</tr>
<tr>
<td>Frameshift:1-BP DEL, 245G
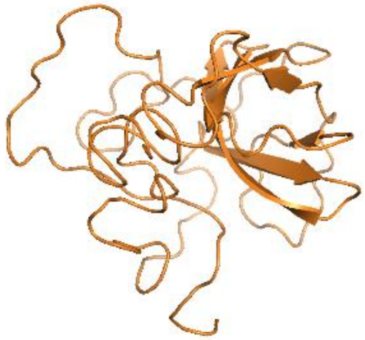
Electrostatic Potential: 1-BP DEL, 245G
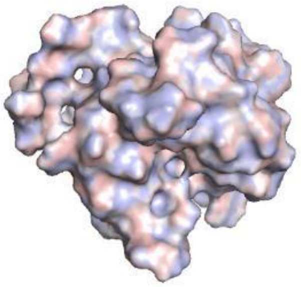</td>
<td>C-score=-1.28
TM-score = 0.56±0.15
RMSD = 7.7±4.3Å

Z-Score: -3.87
Ramachandran plot
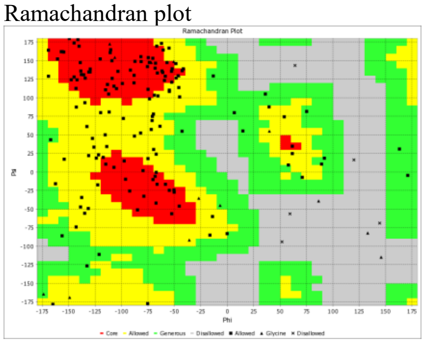</td>
<td>Structure superimposition: KLK4 WT & 1-BP DEL, 245G*
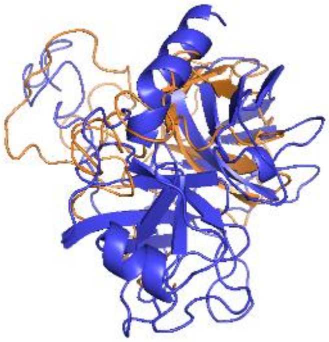
RMSD = 2.025 (971 atoms)</td>
</tr>
<tr>
<td colspan="3">* = Structural change observed (RMSD => 1.5). Colour Scale for Electrostatic Potential 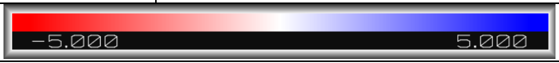</td>
</tr>
</table>
